# Significance of host antimicrobial peptides in the pathogenesis and treatment of acne vulgaris

**DOI:** 10.3389/fimmu.2024.1502242

**Published:** 2024-12-18

**Authors:** Agata Lesiak, Paulina Paprocka, Urszula Wnorowska, Angelika Mańkowska, Grzegorz Król, Katarzyna Głuszek, Ewelina Piktel, Jakub Spałek, Sławomir Okła, Krzysztof Fiedoruk, Bonita Durnaś, Robert Bucki

**Affiliations:** ^1^ Institute of Medical Science, Collegium Medicum, Jan Kochanowski University in Kielce, Kielce, Poland; ^2^ Department of Medical Microbiology and Nanobiomedical Engineering, Medical University of Białystok, Białystok, Poland; ^3^ Independent Laboratory of Nanomedicine, Medical University of Białystok, Białystok, Poland; ^4^ Department of Otolaryngology, Holy-Cross Oncology Center of Kielce, Head and Neck Surgery, Kielce, Poland; ^5^ Department of Clinical Microbiology, Holy-Cross Oncology Center of Kielce, Kielce, Poland

**Keywords:** *Cutibacterium acnes*, acne vulgaris, antimicrobial peptides, skin dysbiosis, inflammation

## Abstract

Acne vulgaris (AV) is a chronic inflammatory condition of the pilosebaceous units characterized by multiple immunologic, metabolic, hormonal, genetic, psycho-emotional dysfunctions, and skin microbiota dysbiosis. The latter is manifested by a decreased population (phylotypes, i.e., genetically distinct bacterial subgroups that play different roles in skin health and disease) diversity of the predominant skin bacterial commensal - *Cutinbacterium acnes*. Like in other dysbiotic disorders, an elevated expression of endogenous antimicrobial peptides (AMPs) is a hallmark of AV. AMPs, such as human β-defensins, cathelicidin LL-37, dermcidin, or RNase-7, due to their antibacterial and immunomodulatory properties, function as the first line of defense and coordinate the host-microbiota interactions. Therefore, AMPs are potential candidates for pharmaceutical prophylaxis or treating this condition. This study outlines the current knowledge regarding the importance of AMPs in AV pathomechanism in light of recent transcriptomic studies. In particular, their role in improving the tight junctions (TJs) skin barrier by activating the fundamental cellular proteins, such as PI3K, GSK-3, aPKC, and Rac1, is discussed. We hypothesized that the increased expression of AMPs and their patterns in AV act as a compensatory mechanism to protect the skin with an impaired permeability barrier. Therefore, AMPs could be key determinants in regulating AV development and progression, linking acne-associated immune responses and metabolic factors, like insulin/IGF-1 and PI3K/Akt/mTOR/FoxO1 signaling pathways or glucotoxicity. Research and development of anti-acne AMPs are also addressed.

## 1 Introduction

In 2010, acne vulgaris (AV) was in the top 10 most prevalent diseases worldwide, affecting 85% of adolescents in Westernized populations (1). Clinically, AV (ICD-11, ED80; according to the International Classification of Diseases 11th Revision; https://icd.who.int/) is considered as a chronic and multifactorial inflammatory disease of the pilosebaceous unit (PSU), i.e., a structure composed of the hair follicle, sebaceous gland, and arrector pili muscle, and associated with excessive sebum production (hyperseborrhea). However, from a physiological perspective, AV is perceived as a metabolic syndrome (MetS) of the sebaceous follicle, and like obesity, type 2 diabetes (T2D), or insulin resistance, belongs to the mechanistic target of rapamycin complex 1 (mTORC1)-driven diseases or so-called ‘Western civilization diseases’ (2, 3). mTORC1, as part of insulin/insulin-like growth factor-1 (IGF1) signaling pathways, is a key regulator of lipid and energy metabolism. Western dietary model, particularly a high hyperglycemic load and dairy product intake, contributes to AV development by overstimulating these pathways (2–4). Microbiologically, AV is a condition of imbalanced microbial skin colonization, i.e., dysbiosis, specifically by *Cutibacterium acnes* (5). Therefore, complex reciprocal interactions between dysregulated (i) lipid metabolism of the sebaceous gland (manifested as hyperseborrhea), (ii) hormonal homeostasis (linked with follicular hyperkeratinization, (iii) microbial skin colonization (specifically by *C. acnes*), and (iv) immune responses, are behind AV pathomechanism. Overall, these factors contribute to the formation of acne lesions, ranging from non-inflammatory comedones to inflammatory papules, pustules, and nodules (2).

**Figure 1:**
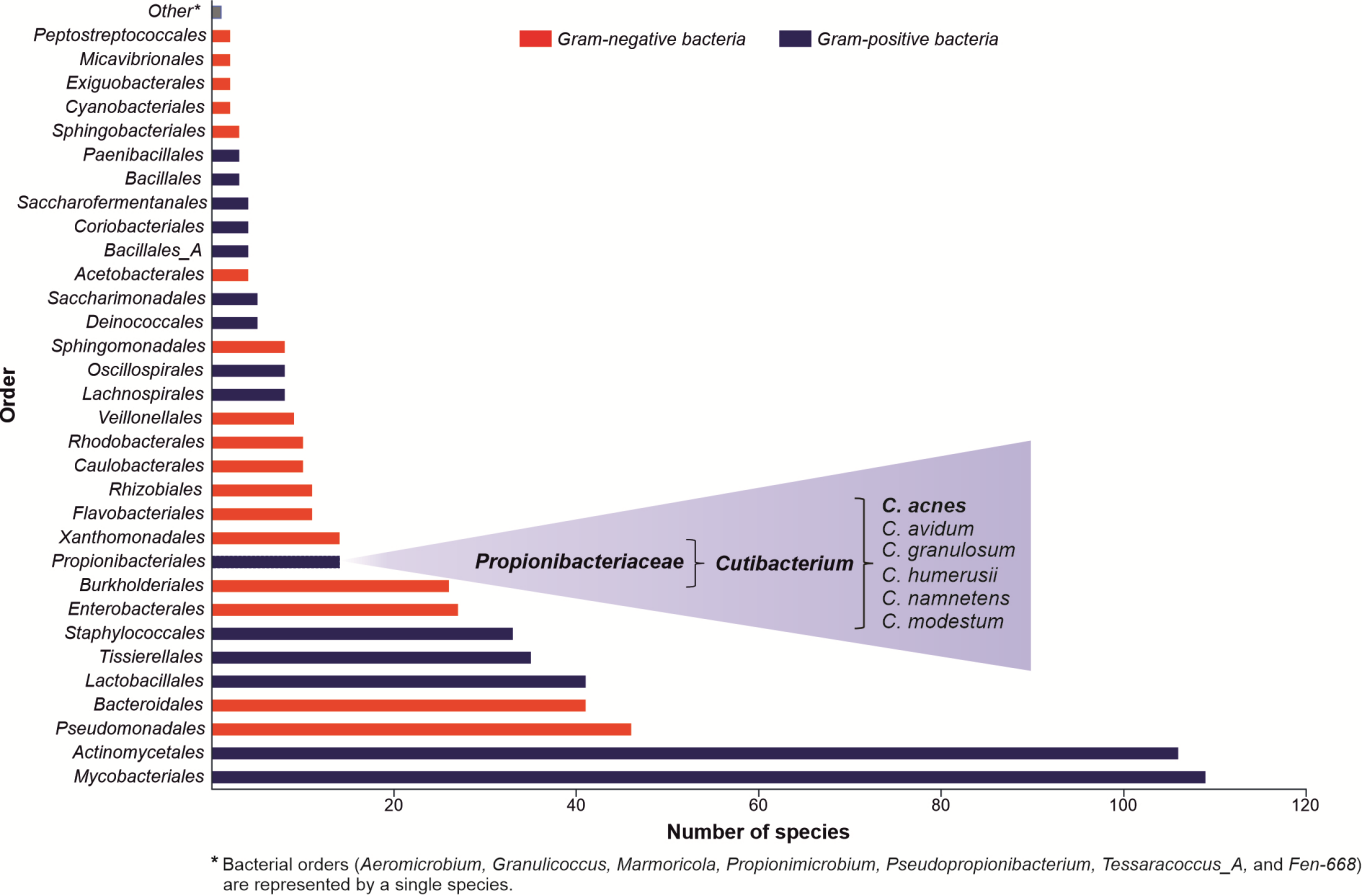
Composition of the skin microbiota. The graph was created based on data collected by the Skin Microbial Genome Collection (SMGC) project. The figure’s information summarizes the published experimental work results (6).

The skin’s epidermis is a physical, chemical, and immune barrier against external infectious and non-infectious insults, also serving as an ecological niche for various microorganisms, collectively known as skin microbiota (**Figure 1**) (7, 8). In between 10^4^ - 10^6^ of bacteria inhabit each square centimeter of the skin (9), which represent 13 phyla with 622 prokaryotic species, where *Actinomycetota* (or *Actinobacteria*) (37.5%), *Proteobacteria* (25.4%), Firmicutes (25.1%), and *Bacteroidota* (8.8%) are the predominant ones (6). Their composition and diversity vary significantly between subjects and across sites, with sebaceous sites dominated by lipophilic *Cutibacterium* species (*Actinomycetota*). In contrast, bacteria that thrive in humid environments, such as *Staphylococcus* (*Firmicutes*) and *Corynebacterium (Actinobacteria*) species, are preferentially abundant in moist areas. The dry regions show the highest diversity, with variable colonization of the four main phyla.

**Figure 2:**
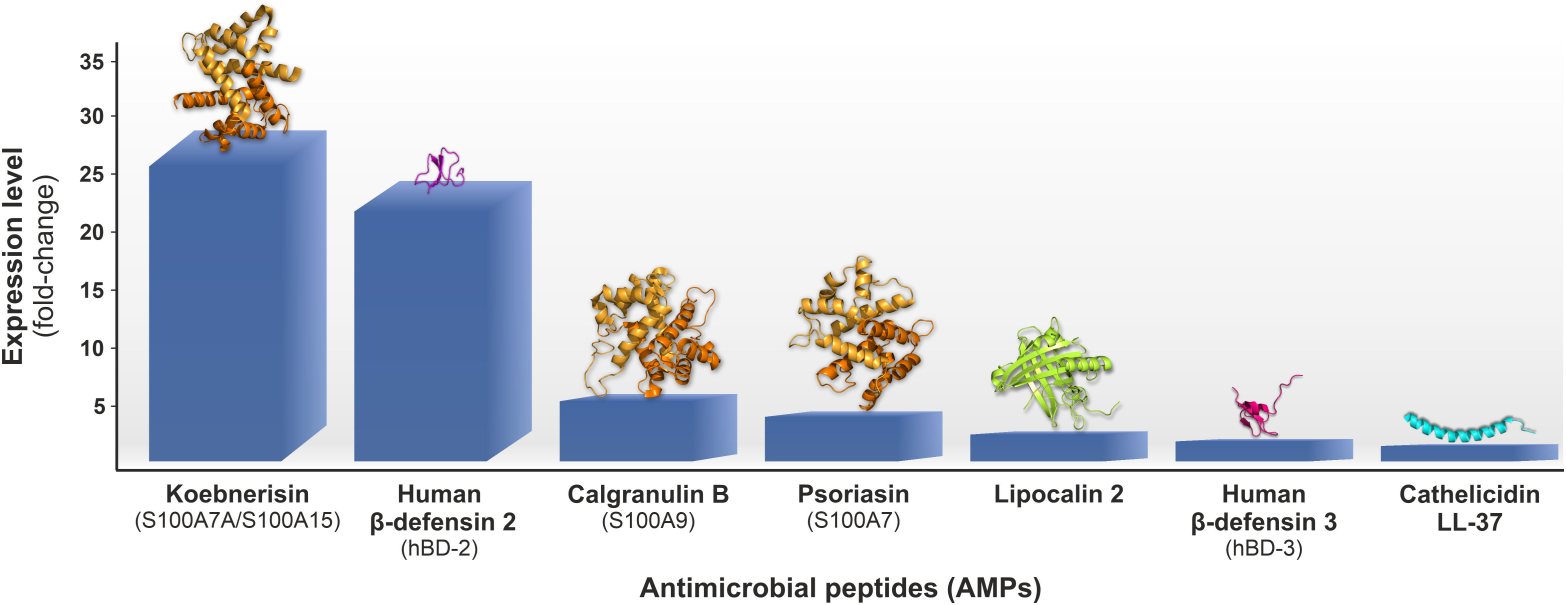
Antimicrobial peptides (AMPs) genes upregulation fold change (AV involved *vs.* non-involved skin) estimated by the microarray transcriptomic analysis of biopsies from early inflammatory AV lesions, i.e., comedones shading into small red papules, collected from twenty subjects with moderate to severe AV reported by Kelhälä et al. (21); 3D protein models were obtained from RCSB Protein Data Bank (https://www.rcsb.org).

The skin microbiota coexists in a symbiotic or commensal relation with the human host, i.e., in a eubiosis state, and acting as a colonization resistance barrier supports a nonspecific skin defense system. Specifically, *C. acnes* and *Staphylococcus epidermidis* are critical for skin homeostasis (10). Dysbiosis, a condition of imbalanced microbiota manifested by qualitative and/or quantitative changes in its composition, is a hallmark of several skin disorders, including AV (5, 9, 11–13). Several mechanisms of *C. acnes-*induced acne aggravation have been proposed, such as augmentation of lipogenesis, alteration of lipid composition in sebum, or exaggeration of host immune responses (5, 14). Multiple innate and adaptive immune mediators mediate the latter, especially an abnormal production of endogenous antimicrobial peptides (AMPs), a hallmark of various dermatoses, such as rosacea, psoriasis, atopic dermatitis, and AV (15–20). According to the transcriptomic analysis of AV lesions, genes encoding AMPs belonging to human β-defensins (hBDs) and S100 family proteins are among the ten most overexpressed ones (**Figure 2**) (21, 22). AMPs are small, mainly cationic peptides with potent antimicrobial and immunomodulatory properties; hence, they are also known as host-defense peptides (HDPs). AMPs contribute to skin homeostasis, serving as (i) the first line of defense against microbial invasion, (ii) coordinators of host-microbiota interactions, and (iii) agents promoting skin integrity and regeneration (23). Specifically, due to the sequestration of pro-inflammatory bacterial factors, such as lipooligosaccharide (LPS) or lipoteichoic acids (LTAs), and suppression of pro-inflammatory cytokines secretion, AMPs act as sensors of microbial load and control microbiota-induced immune defense. However, since various bacterial species- or strain-specific factors, e.g., *C. acnes* virulence factors, can trigger the production of AMPs, an imbalanced proportion or relative abundance of specific bacterial species in skin microbiota or strains may cause an uncontrolled inflammatory response (15–20).

The use of antibiotics, such as clindamycin or macrolides, in treating AV leads not only to the emergence of antibiotic-resistant strains of *C. acnes* but also to other skin commensals. For example, ~30% of *S. epidermidis* isolates from AV patients show resistance to erythromycin, roxithromycin, and clindamycin (24). Therefore, AMPs are promising candidates for novel anti-AV therapies. However, understanding the relationship between AMPs and eubiotic/dysbiotic skin conditions, as well as the host metabolic, hormonal, and genetic factors associated with AV pathophysiology, is essential for developing safe and efficient AMPs-based medications against AV and other dermatoses (25, 26). Here, we summarize the current knowledge regarding the significance of AMPs in AV pathomechanism and progress in research and development of anti-acne AMPs.

## 2 AMPs: structure, characterization, and mechanism of action against bacteria

Antimicrobial peptides (AMPs) are cell membrane-targeting, low-molecular-weight molecules with fewer than 100 amino acid residues, characterized by a broad-spectrum activity against various bacterial, fungal, parasitic, and viral agents (27, 28). AMPs are a part of innate immunity and the first line of defense against microbial pathogens, while prokaryotes use them for interspecies competition, e.g., competitive exclusion (29). Additionally, AMPs regulate the host’s pro-inflammatory and anti-inflammatory responses, either with infectious and non-infectious etiology, as well as a variety of cellular processes, such as chemotaxis, autophagy, apoptosis, cell differentiation, or wound healing (29). Hence, AMPs commonly function as ‘alarmins’ or damage-associated molecular patterns (DAMPs), i.e., molecules produced by injured or dying cells that initiate signaling pathways responsible for various physiological and pathological processes. Thus, the term host defense peptides (HDPs) has emerged to encompass their pleiotropy (30).

The secondary structure divides AMPs into (i) α-helical- (e.g., cathelicidins), (ii) β-sheet- (e.g., human α- and β-defensins) or (iii) αβ-containing peptides (e.g., θ-defensins), and (iv) elongated or loop and rich in proline, tryptophan or glycine AMPs (e.g., indolicidin) (31). In aqueous solutions, the conformation of α-helical AMPs is disordered and adopts an amphipathic helical structure upon contact with biological membranes. This process compromises their integrity and increases permeability, which ultimately causes cell death (32). Several factors influence AMPs action, including peptide size, amino acid sequence, charge, conformation, hydrophobicity, and amphipathic characteristics. The biological activity of AMPs depends on various parameters, including peptide size, amino acid sequence, charge, conformation, hydrophobicity, and amphipathic nature.

Despite their distinct origin and various structural and physicochemical features AMPs share common characteristics (33). The vast majority of AMPs are positively charged (charge from +2 to +11) and composed of 5 to 100 amino acids oligopeptides, rich in leucine, glycine, and lysine (>8%), but not in methionine and tryptophan (<2%) (34). Thus, they are commonly known as cationic antimicrobial peptides (CAMPs). The electrostatic interactions between positively charged peptides and negatively charged bacterial surfaces, e.g., lipopolysaccharides (LPSs) or teichoic acids (TAs), are responsible for their antimicrobial activity. Moreover, AMPs exhibit an amphipathic structure due to hydrophobic and hydrophilic regions, which dictates their structural flexibility and, along with hydrophobicity, determines the membranolytic properties of these compounds and selective toxicity toward microbial cells (35).

Three distinct processes are involved in the production of AMPs: (i) classical ribosomal synthesis, (ii) non-ribosomal synthesis, and (iii) proteolytic processing. The former is utilized to synthesize human β-defensins (hBds) or histatins. In contrast, the non-ribosomal synthesis is typical for bacteria, and it is performed by non-ribosomal peptide synthetases, allowing for the incorporation of non-proteinogenic amino acids into AMPs. Several protein modifications, such as hydroxylation, glycosylation, and cyclization, are also common in AMPs synthesized by this pathway. Finally, certain AMPs, so-called ‘mystery peptides’, can be formed through the proteolytic cleavage of larger proteins with different functions (36). In addition, many AMPs are produced as inactive precursors, requiring proteolytic cleavage to gain functional activity.

The expression of AMPs can be constitutive or inducible. Constitutively expressed AMPs accumulate in high concentrations as inactive precursors in cell granules and are released locally at infection and/or inflammation sites. Inducible AMP expression is triggered by microbial-associated-molecular patterns (MAMPs) and host immune effector factors, such as cytokines (37).

The electrostatic interactions between positively charged peptides and negatively charged bacterial surfaces, e.g., LPSs or TAs, are responsible for their antimicrobial activity. In eukaryotic cell membranes, sphingomyelin and phosphatidylcholine due to their neutral charge at physiological pH, prevent interactions with AMPs. Hence, membrane lipid composition substantially affects the interaction between AMPs and microbial cell envelopes, determining their specificity and activity toward microbial cells. For instance, the cell wall of Gram-positive bacteria, such as *C. acnes*, comprises multiple layers of peptidoglycan (murein), 40-80 nm in diameter, stabilizing bacterial cell shape. Peptidoglycan is a polymer of the disaccharide N-acetylglucosamine and N-acetylmuramic acid cross-linked by peptide bridges with teichoic and lipoteichoic acids (LTAs), linked to N-acetylmuramic acid and membrane lipids, respectively (38). In addition, Gram-positive bacteria have more negatively charged phosphatidylglycerol with saturated, unsaturated, and branched fatty acids than Gram-negative species (39). Although plasma membrane phospholipid content in Gram-positive bacteria varies by species., a high concentration of phosphatidylglycerol and its derivatives, including lysyl phosphatidylglycerol, cardiolipins, and phosphatidylethanolamine, is characteristic of this group (40).

Subsequently, AMPs are classified as ‘membrane-acting’ and ‘non-membrane-acting’ peptides. The former AMPs disrupt microbial membranes, leading to cell death by osmotic shock via (i) the barrel stave mode., (ii) the carpet model, or (iii) the toroidal pore model (31). In contrast, ‘non-membrane-acting peptides’ can penetrate membranes without damaging them and target basic metabolic processes like protein or nucleic acid synthesis and metabolic activity (41). AMPs can also inhibit bacterial cell wall synthesis by binding with lipid II, a glycolipid precursor for this process, or by inhibiting its formation, e.g., by a lipoglycopeptide rhamoplanin (29, 42). Likewise, the biosynthesis of TAs and LTAs AMPs can be affected by AMPs (43).

Due to the cationic and amphiphilic nature of AMPs, they can directly interact with cell membranes with high anionic phospholipid content, accumulating continuously at the membrane surface and inducing structural or conformational changes (44). In sufficient concentrations, AMPs increase cell membrane permeability, breaking the membrane and releasing cellular content (45). In general, the cell membrane-directed activity of AMPs is determined by membrane structural/conformational changes and the peptide-lipid ratio (46). When the latter is high, AMPs gain access to the membrane’s hydrophobic interior, leading to cell death. Otherwise, AMPs remain stacked in a parallel orientation to the cell membrane’s surface (47).

After initial electrostatic and/or hydrophobic interactions, AMPs self-organization on the cell membrane adapts one of the aforementioned models (48, 49). In the barrel-stave pore model, AMPs initially oriented parallel to the cell membrane are inserted perpendicular to the lipid bilayer, forming trans-membrane channels through aggregation and conformational change. The amphipathic structure, α-helical or β-sheet, is crucial for pore formation since hydrophobic regions interface with membrane lipids, whereas hydrophilic residues generate channel lumens. A limited number of AMPs, such as protegrin and alamethicin, may exploit this mechanism due to their scant α-helical and β-sheet residues (50). In contrast, the toroidal pore model is not based on peptide-peptide interactions, as peptide helices attach to membrane lipids to form pores, inducing bend deformation in the lipid molecules. In the carpet model, AMPs, after reaching their maximum concentration, cover the entire membrane surface, causing degradation in a surfactant-like manner, which finally degrades the membrane by generating micelles. Several peptides utilize this model, including cathelicidin LL-37, indolicidin, and aureins (47, 48). The aggregate model is similar to the carpet mechanism in that AMPs are inserted into the cell membrane after they reach a threshold concentration. This interaction triggers a spatial change in the AMPs, allowing micelle-like complexes to form with the lipids and transverse the lipid bilayer in a peptide-lipid complex. These transmembrane aggregates of lipids, AMPs, and water can create channels for ions and intracellular content leakage, causing cell death. This mechanism also allows AMPs to be delivered into the cell, resulting in intracellular killing (51). Multiple intracellular processes can be affected by AMPs, including protein biosynthesis, nucleic acid synthesis, or cell division (52). AMPs binding to nucleic acids results in conformation changes followed by inhibition of DNA, RNA, and protein synthesis utilizing a mechanism similar to histone-DNA interactions. For instance, buforin II and indolicidin interfere with nucleic acids (52). The latter, for example, has a high affinity for the double-stranded binding of ds[AT], ds[CG], ds[AG] and a lower affinity for ds[GT] as well as prevents DNA relaxation via inactivation of DNA topoisomerase I (53, 54). In contrast, human neutrophil peptide-1 (HNP-1) inactivates the DNA damage response system, inducing programmed bacterial cell death, by blocking the interaction of RecA with single-stranded DNA (ssDNA) (55). Similarly, proline-rich AMPs interfere with bacterial protein folding by inhibiting the bacterial heat shock protein (DnaK), and human alpha-defensin 5 (HD5) inhibits bacterial cell division (56, 57).

## 3 Acne vulgaris pathomechanism

Clinically, skin lesions, such as open comedones (blackheads) and closed comedones (whiteheads), papules, pustules, cysts, and nodules, on the face, back, chest, and other sebaceous gland-rich areas are typical manifestations of AV (58), observed in ~80% of young adults and adolescents (59, 60). Open and closed comedones are generally benign and non-inflammatory, whereas papular and pustular lesions are mild to moderately inflammatory, and nodules represent the most severe type of lesions. However, this classification may not accurately reflect the role of inflammation in AV pathogenesis, which appears to be significant at all stages of acne development, perhaps even before comedo formation (61).

On the transcriptome level, depending on the study, AV upregulates 211 or 904 genes and downregulates 18 or 395 genes (21, 22). The upregulated genes are primarily involved in inflammation, including mediators of innate/adaptive immunity and matrix remodeling processes (22). The former genes include interleukins 8 (IL-8) and 1 (IL-1), chemokine (C-C motif) ligands (CCLs), cytokines associated with the IL-17/Th17 pathway activation, such as IL-23, IL-6, and transforming growth factor β (TGF-β), as well as various AMPs (21). In fact, genes encoding specific AMPs, i.e., S100 family proteins and hBDs, were among the top ten overexpressed in both studies (21, 22). For instance, in the study by Kelhälä et al., the gene encoding S100A15 (S100A7A) protein, also known as koebnerisin, was the most upregulated one, with a fold change value of 29 (**Figure 2**) (21). Furthermore, the genes encoding matrix metalloproteinases (MMPs) and protease inhibitors (PIs), like elafin (peptidase inhibitor 3, PI3) or skin-derived antileukoprotease (SKALP), represented the leading overexpressed matrix remodeling factors (21, 22).

**Figure 3:**
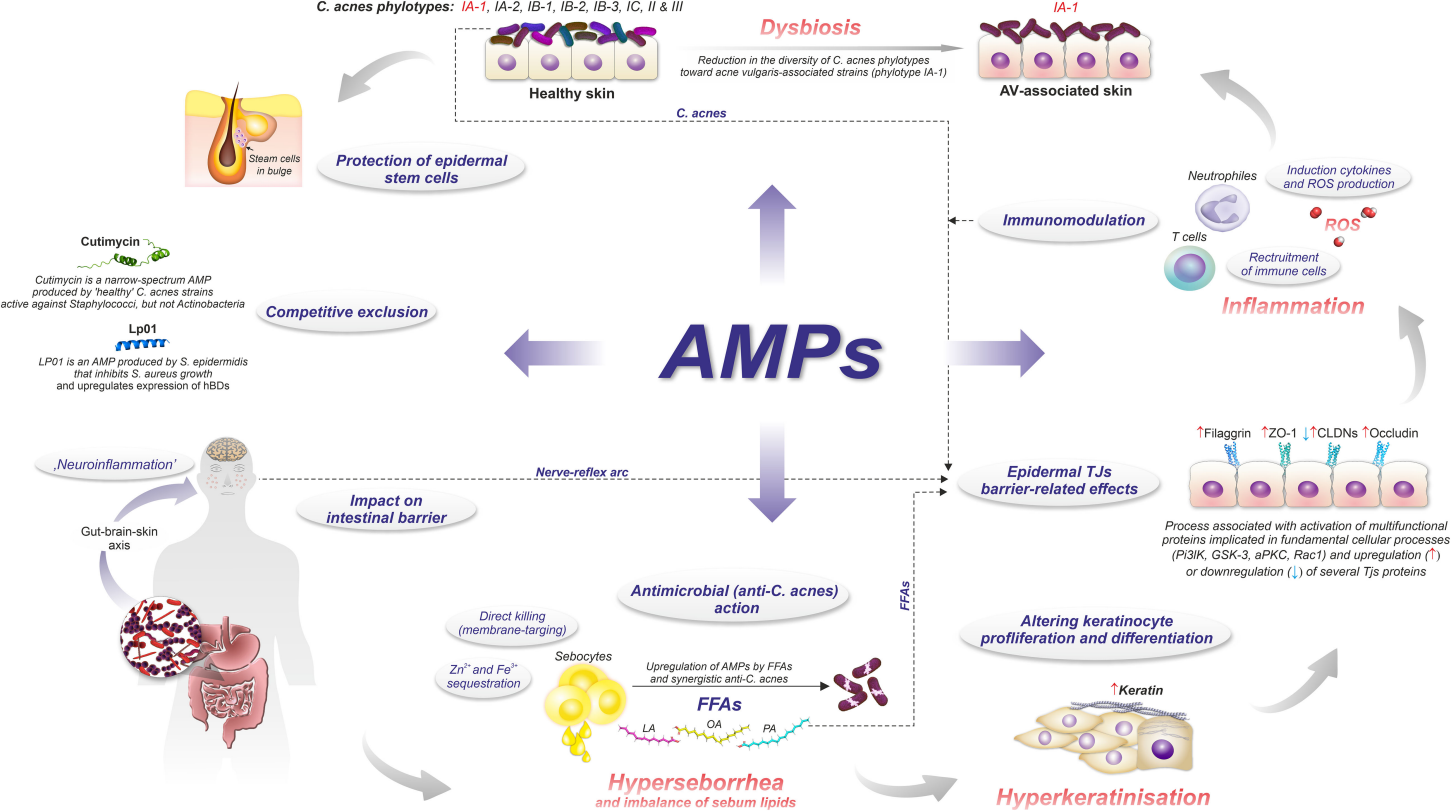
Role of AMPs in AV pathomechanism.

**Figure 4:**
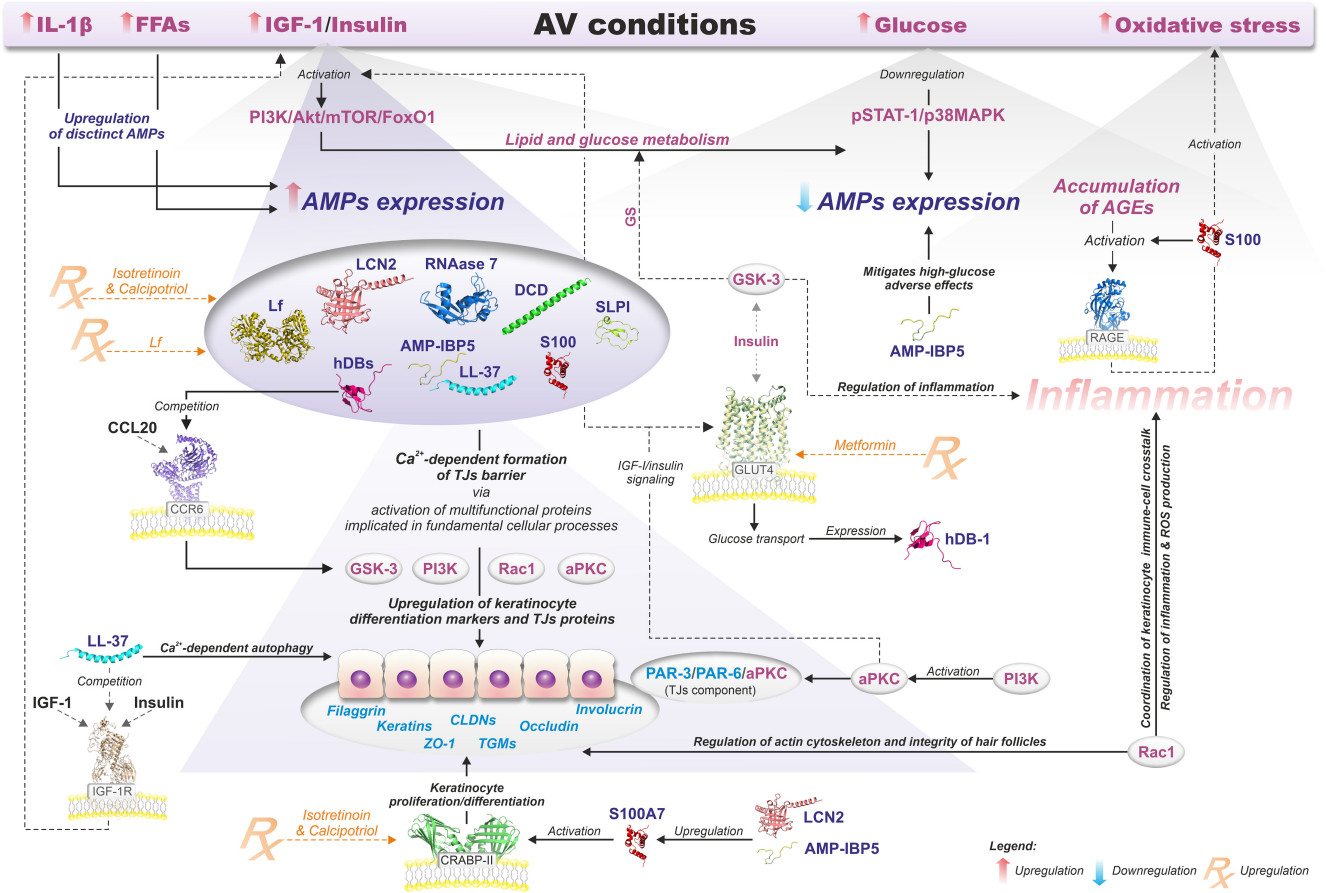
Relationship between AMPs and the modulation of epidermal TJs barrier function in AV metabolic background context. Details are discussed in the text with the use of citations. All information presented in the figure summarizes the results of the published experimental work (19, 63–112).

In general, the pathophysiology of AV involves the sequence of (i) androgen-induced sebum hypersecretion (hyperseborrhea), (ii) hyperkeratinization and hyperproliferation along with abnormal keratinocyte differentiation in hair follicles, (iii) dysbiosis of skin microbiota characterized by a decrease in population (phylotypes, i.e., genetically distinct bacterial subgroups that play different roles in skin health and disease) diversity of lipophilic skin commensal *C. acnes*, and (iv) aberrant host inflammatory response (**Figure 3**) (62). Recently, a mechanical skin barrier impairment has also been proposed as a novel explanatory variable of AV pathomechanism (63). Notably, AMPs appear to be key players in the enhancement of impaired, e.g., by *C. acnes*, skin barrier function through the activation of various fundamental cellular proteins, such as phosphoinositide 3-kinases (PI3K), glycogen synthase kinase-3 (GSK3), atypical protein kinase C (aPKC), and Ras-related C3 botulinum toxin substrate 1 (Rac1) (**Figure 4**) (63).

### 3.1 Androgen- induced sebum hypersecretion

The androgen-induced hyperseborrhea is mediated via overstimulation of insulin, insulin-like growth factor (IGF-1), kinase Akt, and mTORC1 signaling pathways (insulin/IGF-1/Akt/mTORC1) (113–116). Accordingly, a decreased responsiveness of the sebaceous glands to insulin-mediated signals, i.e., (insulin resistance), is compensated by the upregulation of IGF-1, which stimulates the production of androgens (115).

Activation of mTORC1 causes the accumulation of triacylglycerols inducing adipo- and lipogenesis and inhibiting lipid catabolism, e.g., lipolysis and β-oxidation. Moreover, it promotes sebaceous lipogenesis via the PI3K/Akt/FoxO1/mTORC1 pathway and induction of the sterol regulatory element binding protein 1 (SREBP1) (3, 64, 65). In this process, Akt-mediated phosphorylation of nuclear forkhead transcription factors O1 and O3 (FoxO1 and FoxO3) initiates their extrusion into the cytoplasm, followed by the stimulation of lipogenic and proinflammatory transcription factors, such as androgen receptor (AR), sterol regulatory element-binding transcription factor 1 (SREBF1), peroxisome proliferator-activated receptor γ (PPARγ), and signal transducer and activator of transcription 3 (STAT3). At the same time, FoxO1-dependent expression of GATA binding protein 6 (GATA6) is decreased. GATA6 is a transcription factor vital for keratinocyte homeostasis, which prevents hyperkeratinization of the infundibulum. Accordingly, its downregulation in PSUs of AV patients has been reported (117). Additionally, the Akt-mediated phosphorylation of mouse-double minute 2 (MDM2) promotes the degradation of the transcription factor p53, resulting in decreased p53-mediated expression of FoxO1, FoxO3, and other p53 target genes (117).

Furthermore, hyperglycemic carbohydrates and insulinotropic dairy products also provoke the insulin/IGF-1/Akt/mTORC1 signaling, linking AV with a Western diet and the gut microbiota via gut-skin and gut-brain-skin axes (14, 118, 119). For instance, in animal models, long-term stress induces dysbiosis of the gut microbiota, e.g., characterized by a deficit of probiotic genera like *Lactobacillus* and *Bifidobacterium* (64, 65, 120). The intestinal microbiota-derived metabolites, such as short-chain fatty acids (SCFAs), could modulate innate and adaptive immunity through multiple signaling pathways, including mTOR (121, 122). Likewise, the gut microbiota, through the production and release into the blood neurotransmitters, such as acetylcholine, serotonin, norepinephrine, and increasing intestinal permeability, may contribute to stress-induced acne exacerbation by gut-brain-skin axis (14, 123). Specifically, emotional stresses appear to affect the function of PSUs, contributing to the development and/or aggravation of pre-existing acne via stimulation of (i) hormone production, (ii) neuropeptides, such as corticotropin-releasing hormone (CRH), (iii) melanocortins, such as alpha-melanocyte-stimulating hormone (α-MSH) and adrenocorticotropic hormone (ACTH), and substance P, as well as (iv) proinflammatory cytokines (124). Hormones, for example, via the CRH receptor 1, enhance sebaceous lipid synthesis and the release of IL-6 and IL-8 by sebaceous glands. In addition, CRH stimulates the secretion of adrenocorticotropic hormone (ACTH), hence, the production of dehydroepiandrosterone by the adrenal glands (125, 126). Furthermore, CRH activates the hypothalamic-pituitary-adrenal (HPA) axis resulting in cortisol release from the adrenal glands. Cortisol as a potent insulin-antagonistic hormone inhibits insulin secretion, promotes insulin resistance and hyperglycemia (127). Increased levels of cortisol can also suppress hypothalamic-pituitary-gonadal (HPG) axis and gonadotropin-releasing hormone (GnRH) secretion leading to lowers androgen production. Similarly, Substance P participates in the regulation of glucose metabolism via insulin signaling-associated pathways, and in rats its intravenous administration leads to hypoinsulinemia, hyperglucagonemia, and subsequently to hyperglycemia (128, 129). Melanocortins regulate glucose homeostasis via central nervous system pathways, primarily in the hypothalamus, via melanocortin receptors (MC1R–MC5R) (130). Activation of these receptors, especially MC4R, has been associated with improved insulin sensitivity and glucose homeostasis (130). Moreover, insulin-dependent regulation of intracellular glucose levels significantly impacts the expression of hBDs (**Figure 4**) (66).

### 3.2 Hyperkeratinization

Apart from hyperseborrhea *per se*, an imbalanced proportion of sebum-specific lipids plays a pivotal role in AV pathomechanism. The sebum comprises a diverse array of lipids, such as triglycerides and free fatty acids (FFAs) (40%–60%), wax esters (20%–30%), squalene (10%–20%), and cholesterol and its esters (2%–10%) (115). Accordingly, sebum of patients with AV is deficient in essential FFAs, like linoleic acid (LA), and relatively abundant in proinflammatory lipids, such as monounsaturated fatty acids (MUFAs) and lipoperoxides. The latter are products of squalene peroxidation and significantly contribute to the hyperkeratinization of the PSUs, inducing keratinocyte proliferation/differentiation and can initiate an inflammatory response, activating PPARs-dependent signaling pathways and triggering the secretion of pro-inflammatory cytokines (61, 131, 132). Likewise, palmitic acid (PA) is a powerful stimulator of the NLRP3 inflammasomes in macrophages (122). In contrast, LA has anti-inflammatory properties, and in human monocytes, it can suppress NF-κB signaling by a PPARγ-dependent mechanism (133). In addition, β-oxidated derivatives of linoleic acid (LA) serve as precursors for acetyl-CoA - a key metabolite for the biosynthesis of lipids. Therefore, a deficiency of LA in the hair follicle disturbs the composition of squalene, wax esters, and sphingolipids. Furthermore, FFAs, by promoting the expression of human β-defensin 2 (hBD-2) in sebocytes, may contribute to the skin’s natural barrier (67). Similarly, sphingolipids, such as ceramides, as essential epidermal barrier components controlling transepidermal water loss and directing processes of keratinocyte proliferation, differentiation, and apoptosis, are implicated in numerous dermatoses (134). In AV, sphingolipid deficiency stimulates follicular hyperkeratosis and abnormal desquamation of epithelium, resulting in pores clogging and comedone formation. Overall, sebum-rich and hypoxic conditions of comedone act as a dysbiotic factor that promotes their colonization by anaerobic and lipophilic species, like specific phylotypes of *C. acnes* (see below). This shift in the *C. acnes* population toward so-called ‘acne-associated’ phylotypes (or strains), characterized by enhanced inflammatory potential compared to the ‘healthy’ phylotypes, ultimately leads to intra-species dysbiosis (10, 135–138). As an illustration, Dagnelie et al. reported that the innate immune response of healthy skin explants exposed to a mix of three *C. acnes* phylotypes was weaker when compared to their individual applications. Therefore, the diversity of the *C. acnes* population appears to play a vital role in maintaining the cutaneous microbiota in eubiosis and may serve as a biomarker of healthy skin (139).

### 3.3 Overgrowth of *C. acnes*

*C. acnes* (formerly *Propionibacterium acnes*) is a lipophilic, slow-growing, aerotolerant, anaerobic, rod-shaped Gram-positive bacterium important for human health as a predominant cutaneous and mucous membrane commensal, as well as an opportunistic pathogen, mainly involved in device- and biofilm-associated infections (140).

**Figure 5:**
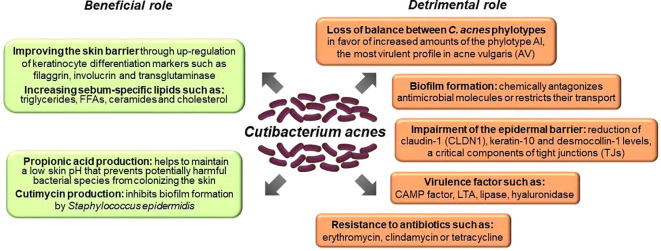
Role of *C. acnes* in skin health and AV pathomechanism.

As a vital member of the skin microbiota, *C. acnes* contributes to skin homeostasis via (i) lipid metabolism (ii) skin acidification, (iii) colonization resistance and niche competition with other skin microbiota (competitive exclusion), (iv) antioxidant effects, and (v) immunomodulatory properties (**Figures 3**, **5**) (10, 11, 139, 141–143). *C. acnes* substantially raises the amount of several sebum-specific lipids, such as triglycerides, FFAs, ceramides, and cholesterol (141). This activity is mediated mainly by its metabolites, like SCFAs, which interfere with the expression of multiple lipid synthesis genes, such as glycerol-3-phosphate-acyltransferase (GPAT), in the PPARα-dependent mechanism (**Figure 3**) (141). Overall, *C. acnes* promotes sebum secretion along with changes in its composition by the formation of FFAs, squalene oxidation, and increasing activity of diacylglycerol acyltransferase, resulting in higher levels of MUFAs and a decrease of LA abundance (136).

*C. acnes*-derived SCFAs contribute to its colonization resistance and competitive strategies. Maintaining skin pH at 5 to 6, mainly by producing propionic acid, prevents its colonization by potentially harmful species, such as *Staphylococcus aureus* or *Streptococcus pyogenes*. SCFAs promote the growth of other commensal species and the activity of pH-dependent lipid-synthesis enzymes (141, 144, 145). Also, the skin barrier is improved by *C. acnes*, through the upregulation of several keratinocyte differentiation markers, such as filaggrin, involucrin, and transglutaminases (146). Conversely, *C. acnes* prevents biofilm formation by *S. epidermidis* and inhibits the growth of Staphylococci, producing an antimicrobial thiopeptide - cutimycin (**Figures 3**, **5**) (142, 147).

On the other hand, *C. acnes*-derived SCFAs under hypoxic conditions inhibit histone deacetylases (HDACs) in human sebocytes and keratinocytes, exerting pro-inflammatory effects due to their enhanced responsiveness to Toll-like receptor-2 (TLR2)-dependent activation by cytokines expression and upregulation of free fatty acid receptors (FFARs), e.g., G protein-coupled receptors (GPRs) (148). Likewise, overstimulation of immune responses by *C. acnes* may lead to the reduction of claudin-1 (CLDN1) expression, which is a critical component of epidermal tight junctions (TJs) and substantially contributes to skin barrier function (146). Specifically, in keratinocyte monolayer cultures, *C. acnes* 889 decreased and increased levels of CLDN1 and CLDN4, respectively. Since CLDN4 is a tightening claudin, these opposite changes in CLDN1 and CLDN4 levels were explained as a compensatory mechanism that counteracts the impairment of the epidermal barrier due to CLDN1 downregulation. Similarly, *C. acnes* extracellular vesicles (EVs) may decrease epidermal keratin-10 and desmocollin through TLR2-dependent signaling (149). These observations suggest that AV pathogenesis may involve additional, beyond immune and inflammatory responses, mechanisms linked to mechanical skin barrier impairment (**Figures 3**, **4**) (63).

### 3.4 Host inflammatory response

*C. acnes* produces numerous enzymes, such as lipases, hyaluronidases, proteases, polyunsaturated fatty acid isomerases, glycosidases, and sialidases, acting as spreading and damage-associated molecular patterns (DAMPs)-inducing factors. As spreading factors, they accelerate extracellular matrix decomposition (ECMs) and, in turn, infiltration of hair follicles by inflammatory cells, such as neutrophils or monocytes, followed by their damage and releasing bacteria, keratin, and sebum into the dermis, initiating the scarring process (150). Accordingly, Trivedi et al. reported that genes implicated in matrix remodeling, such as MMPs and PIs, are among the most upregulated in AV patients (22). *C. acnes* can increase the production of several MMPs, such as MMP-1, MMP-9, and MMP-13, and a correlation between elevated levels of MMP-9 and the number of acne-induced skin lesions, e.g., pustules, has been reported (14, 151, 152). In addition, *C. acnes* can stimulate CD44 and TRL2 signaling pathways due to the degradation of hyaluronic acid (HA) by its hyaluronate lyase (HYL-IA) into fragments acting as ligands these cellular receptors (150). Likewise, pro-inflammatory responses could be triggered by other *C. acnes* virulence factors, such as porphyrins, CAMP (Christie-Atkins-Munch-Peterson) factors, dermatan-sulfate adhesins DsA1 and DsA2, HtaA iron acquisition protein (4). For example, CAMP1 upregulates multiple cytokines by a TLR2-dependent mechanism (153). In contrast, porphyrins contribute to the formation of acne skin lesions, initiating an inflammatory cascade via CD36 activation and stimulation of reactive oxygen species (ROS) production in keratinocytes (50). In addition, *C. acnes* induces secretion of IL-1β, a key inflammatory mediator, via NLRP3 and caspase-1 activation, also implicating inflammasome-mediated inflammation in AV pathogenesis (14). It should be emphasized that IL-1β is a crucial cytokine in AV pathogenesis, severity, and post-acne scar formation (68, 69).

**Figure 6:**
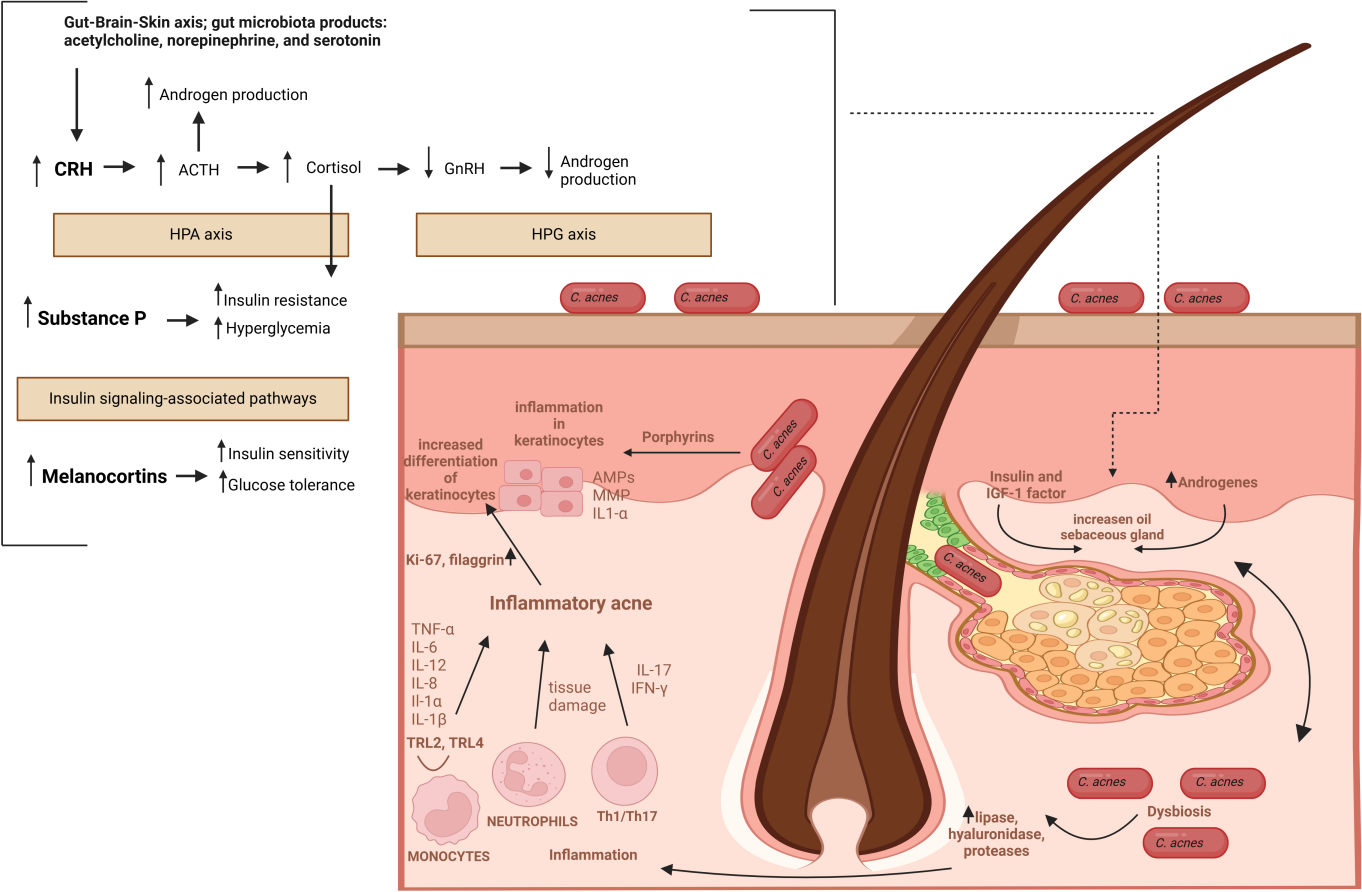
The pathomechanism of AV. Abbreviations: CRH, corticotropin-releasing hormone; ACTH, adrenocorticotropic hormone; HPA axis, hypothalamic-pituitary-adrenal axis; HPG axis, hypothalamic-pituitary-gonadal axis; GnRH, gonadotropin-releasing hormone; MC4R, melanocortin receptor 4.

Finally, *C. acnes* triggers the secretion of Th effector cytokines, such as IL-17 and interferon-γ (INF-γ), through the activation of CD4+ Th lymphocytes, including Th1 and Th17 cells (14, 154). The pathomechanism of AV is summarized in **Figure 6**.

### 3.5 *C. acnes* phylotypes – ‘acne’-associated and ‘healthy’ strains

Phylogenetically, *C. acnes* has been classified into three phylotypes (I-III) with subspecies status, i.e., *C. acnes* subsp. *acnes (I)*, *C. acnes* subsp. *defendens* (II) and *C. acnes* subsp. *elongatum (III)* (14, 155, 156). Furthermore, based on gene sequence typing (ST), the phylotype I was divided into clades IA-1, IA-2, IB, IC (Belfast MLST scheme) or I-1a, I-1b, I-2 (Aarhus MLST scheme) (14, 157), and subsequently subdivided by the whole genome sequencing (WGS) into IA-1, IA-2, IB-1, IB-2, IB-3, IC (14, 158). The *C. acnes* phylotypes exhibit distinct biological properties, including a different composition of the cell wall polysaccharides, lipase activity, patterns of virulence factors, susceptibility to bacteriophages, and varied inflammatory potential; hence, they have different impacts on human health. Specifically, the phylotype IA-1 (or IA-2, depending on the region) *C. acnes* strains (so-called acne-associated *C. acnes* strains) show a significant positive correlation with AV when compared with other, so-called ‘healthy’ *C. acnes* phylotypes (14, 50, 155, 159–161). Therefore, transitioning from a mixed *C. acnes* cutaneous population to one dominated by acne-associated strains is responsible for skin microbiota dysbiosis (10, 135–138).

The acne-associated *C. acnes* strains carry specific virulence genes, and exhibit increased biofilm formation and survival capacity in acne inflammatory milieu (**Figure 5**) (14, 50, 155, 159, 160). For example, the presence of a virulence linear plasmid with a tight adhesion locus associated with biofilm formation is a phylotype I-specific trait (162). Moreover, porphyrins-dependent ROS-related inflammatory response in keratinocytes is promoted explicitly *by* acne-associated *C. acnes* isolates due to the lack of a porphyrin biosynthesis repressor gene (*deoR*) (14, 50, 155, 159, 160). Notably, FFAs, like linoleic acid, significantly suppress ROS activity, highlighting the role of oxidative stress (OS) in AV pathomechanism (70, 71). Acne-associated *C. acnes* strains may also directly modulate keratinocyte proliferation and differentiation by overexpression of IGF-1/IGF-1R, increasing the Ki67 proliferation index, and expression of filaggrin and multiple integrins (α-3, α-6, and vβ-6) in the epidermis (163, 164). They also upregulate IL-10, a pro-inflammatory cytokine associated with chronic inflammation and the development of nodular lesions (139). Furthermore, a high rate of biofilm development by *C. acnes* phylotype IA strains, along with hyperkeratinization and excessive sebum production, causes a blockage of the PSUs (143, 165).

## 4 Role of AMPs in AV pathomechanism

Over 20 AMPs have been linked with skin defense and grouped based on their activity into (i) antimicrobials, (ii) protease inhibitors, (iii) chemokines, and (iv) neuropeptides (23). The human β-defensins, S100 proteins, RNases, cathelicidin LL-37 (LL-37), and dermicidin represent the best-studied skin-derived AMPs (166). In human skin, AMPs are constitutively or inducibly produced by numerous (i) resident cells, such as keratinocytes, sebocytes, sweat glands, and mast cells, as well as (ii) immune system cells, e.g., neutrophils and natural killer cells, recruited in response to injury, inflammation or skin infections (23, 72, 167, 168). Besides the direct antimicrobial activity, the skin-derived AMPs participate in maintaining skin physiological function through (i) coordination of the immune response, (ii) improvement of its permeability, (iii) angiogenesis, and (iv) re-epithelialization. Thus, dysregulated expression of AMPs, e.g., due to injury, infection, or abnormal inflammatory response, is a well-recognized trait in several chronic inflammatory skin diseases, like psoriasis, atopic dermatitis, and rosacea (15, 23, 167, 169). For instance, the upregulation of LL-37 correlates with skin inflammation in rosacea (170). Likewise, deficiency of hBD-2, hBD-3, LL-37, and dermicidin predisposes patients with atopic dermatitis to skin infections (19, 20, 171). On the contrary, cutaneous infections are rare in patients with psoriasis due to the upregulation of LL-37, hBD-2, and S100 proteins (172, 173).

Several studies have revealed overexpression of multiple AMPs in AV patients, such as α- and β-defensins (HNPs and hBDs), S100 proteins, LL-37, RNase 7, lipocalin 2 (LCN2), lactoferrin (Lf), or dermcidin (DCD). In fact, according to transcriptomic studies of AV patients, the genes encoding specific AMPs, i.e., representing S100 family proteins and hBDs, are the top overexpressed ones (21, 22). For instance, in the microarray transcriptomic analysis of biopsies from early inflammatory AV lesions collected from twenty subjects with moderate to severe AV reported by Kelhälä et al., the genes encoding S100A15 (S100A7A) protein and human β-defensin 2 (hBD-2) were the most upregulated ones, respectively with 29- and 25-fold change in expression (**Figure 2**) (21). Also, in the study by Trivedi et al., hBD-2 was the fourth most upregulated gene in inflammatory papule biopsies collected from six AV patients (22).

**Figure 7:**
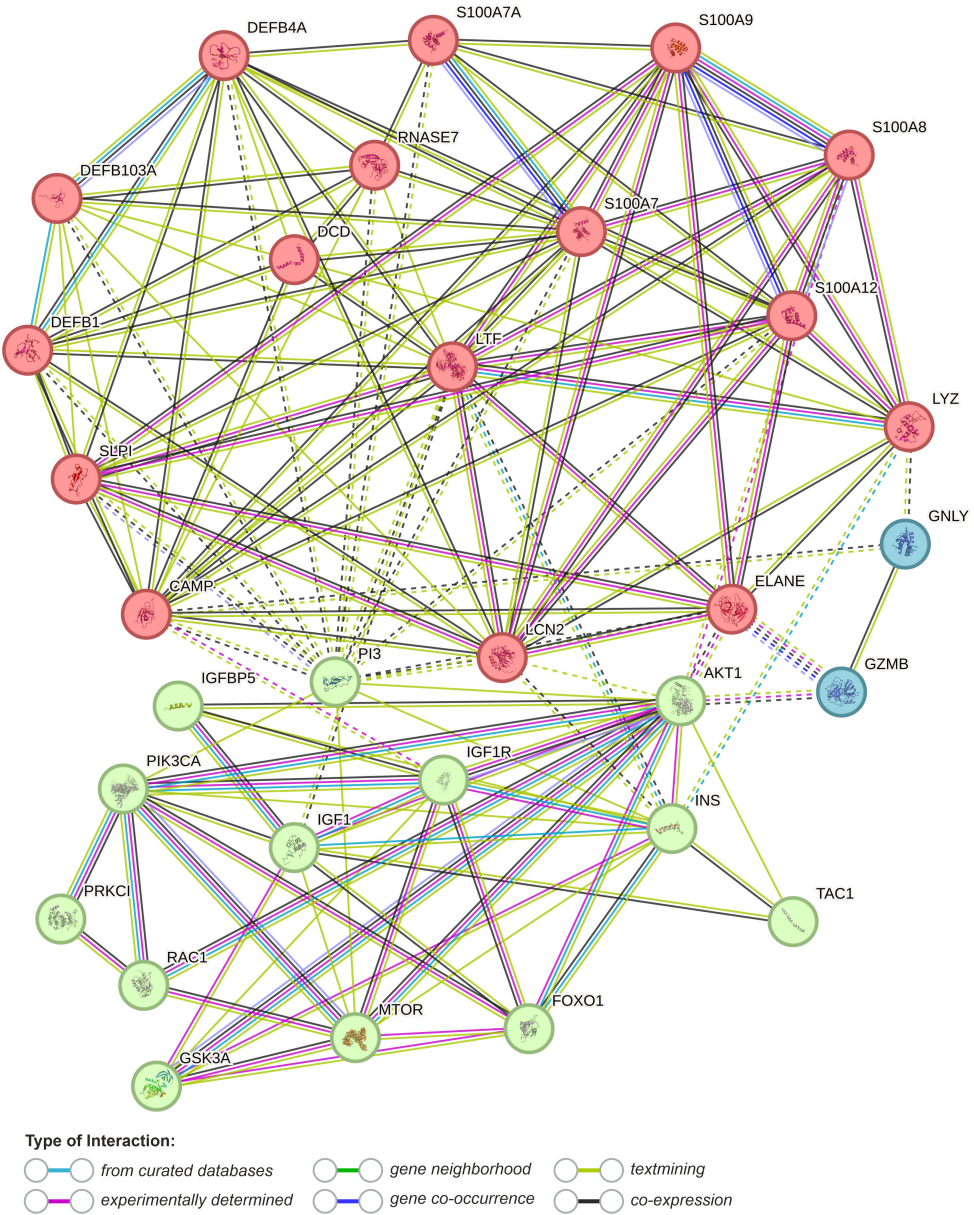
Functional association networks between AMPs and AV metabolic background obtained from String database with cluster analysis (k-means clustering, dotted lines represent edges between clusters) (last accessed 17.03.2024) (177). DEFB1, human β-defensin 1 (hBD-1); DEFB4A, human β-defensin 2 (hBD-2); DEFB103, human β-defensin 3 (hBD-3); S100A7, psoriasin; S100A8, calgranulin A; S100A9, calgranulin B; S100A12, calgranulin C; S100A7A, S100A15 (koebnerisin); SLPI, secretory leukocyte protease inhibitor; PI3, elafin; LTF, lactoferrin; DCD, dermcidin; LYZ, lysozyme; RNASE7, Ribonuclease 7; CAMP, cathelicidin LL-37; LCN2, lipocalin 2 (neutrophil gelatinase-associated lipocalin); GNLY, granulysin; GZMB, granzyme B; ELANE, neutrophil elastase; TAC1, substance B; PI3KCA, phosphoinositide 3-kinase; PRKC1, atypical protein kinase C (aPKC); RAC1, Ras-related C3 botulinum toxin substrate 1; INS, insulin; IGF1 - insulin-like growth factor 1; IFGF1R, insulin-like growth factor 1 receptor; AKT1, RAC-alpha serine/threonine-protein kinase; MTOR, Serine/threonine-protein kinase mTOR; FOXO1, forkhead box protein O1.

The precise function of AMPS in AV pathomechanisms remains to be clarified. However, AMPs (i) antimicrobial, (ii) anti- and (iii) pro-inflammatory, (iv) neuromodulatory (e.g., via gut-brain-skin axis), as well as (v) epidermal TJs barrier-related effects, are considered (**Figure 3**) (15, 17, 63, 166, 169, 174–176). In particular, TJs barrier-related effects of AMPs may shed new light on this process, linking AMPs with AV metabolic (as well as immune and microbial) background via activation of essential for cellular metabolism and signaling proteins, such as phosphoinositide 3-kinases (PI3K), atypical protein kinase C (aPKC), glycogen synthase kinase-3 (GSK3), and Ras-related C3 botulinum toxin substrate 1 (Rac1) (**Figures 3**, **4**, **7**).

For instance, Boronova et al. published an interesting study on the relationship between AMPs and isotretinoin (13-cis retinoic acid, 13-cis RA) treatment in AV patients (178). The authors examined the expression levels of fifteen AMPs in acne skin biopsies across six months of isotretinoin therapy (178). Compared to healthy controls, an increased expression of LL-37, hBD-2, psoriasin (S100A7), koebnerisin (S100A15), RNase 7, lactoferrin, and lysozyme (Lyz) was observed in untreated acne lesions. While dermcidin, granulysin (GNLY), RANTES (CCL5), perforin, CXCL9, and two neuropeptides (substance P and chromogranin B) remained unaffected either in untreated acne patients as well as by isotretinoin treatment. Furthermore, AV patients showed reduced α-defensin-1 (HNP-1) expression levels before treatment. However, this observation is in contradiction to the study by Aidsen et al., who demonstrated a significant reduction in the perivascular and interstitial HNP 1-3 expression of pustular lesions after isotretinoin treatment; hence, the possibility of technical issues was considered (179).

### 4.1 Factors affecting the expression of AMPs

In addition, isotretinoin treatment suppressed the upregulated AMPs to varied degrees, except for lysozyme and RNase 7. However, only LL-37 and koebnerisin (S100A15) returned to baseline levels, indicating their potential as biomarkers of acne treatment efficacy. The continued overexpression of lactoferrin, hBD-2, and psoriasin (S100A7) during isotretinoin treatment implies their involvement in both active and healed (subclinical) AV. Therefore, the authors concluded that their beneficial effects, e.g., anti-*C. acnes* action of hBD-2, may outweigh any pro-inflammatory action, addressing their acne-associated regulation mechanisms. On the other hand, hDBs may perpetuate inflammation by arresting Th17 cells on inflamed sites (**Figure 3**). Accordingly, regulation of Tregs/Th17 responses through TGF-β-dependent generation of Foxp3 is a likely mechanism of isotretinoin anti-acne properties (73, 180). These mechanisms might also be behind the anti-acne action of calcipotriol (a vitamin-D derivative) (17, 74, 75). However, compared to isotretinoin, calcipotriol strongly downregulates psoriasin (S100A7) and koebnerisin (S100A15) while it upregulates LL-37 expression, highlighting the complexity of regulatory mechanisms behind the expression of AMPs in AV (74, 75). Furthermore, isotretinoin therapy did not impact lysozyme and RNase 7, suggesting their, especially RNAse 7, mainly antibacterial rather than pro-inflammatory role in AV pathogenesis. RNase 7 shows high *in vitro* activity against *C. acnes* (LD90 = 4 μM) (16, 178, 181). RNase 7 is secreted by keratinocytes on the skin surface and in PSUs, where it may control microbial colonization (16, 17, 178). For instance, its low expression increases the risk of *S. aureus* cutaneous infection (18). In contrast, lysozyme cannot kill *C. acnes*, which may explain its minor upregulation in acne skin biopsies (178, 181).The expression of AMPs can also be affected by several AV-associated factors, such as FFAs, glucose, insulin, or IGF-1 levels (67, 72, 76, 77). In human sebocytes, FFAs, such as lauric acid, palmitic acid, or oleic acid, can significantly increase the production of hBD-2, but not hBD-1, hBD-3, or LL-37, via CD36 and the NF-κB signaling pathways (67). Furthermore, the anti-*C. acnes* activity of the supernatant from FFA-incubated sebocyte culture can be neutralized by anti-hBD-2, suggesting that this β-defensin is responsible for the antibacterial properties of sebum (174). Notably, *in vitro* hBD-2 at concentrations ≤2.5 μM does not exert anti-*C. acnes* action (78). Whereas in combination with a sublethal dose (25 μM) of lauric acid (but not with palmitic or oleic acid), it shows the dose-dependent killing of *C. acnes*, implicating their synergistic action (67). Therefore, without the context of the inflamed pilosebaceous milieu, the results produced by *in vitro* antimicrobial tests may lead to misleading conclusions regarding the antimicrobial efficiency of individual AMPs (78).

Accordingly, Chronnell et al. correlated the constitutive expression of hBD-1 and hBD-2 across various hair follicle compartments with microbial exposure (174). Specifically, the distal parts of the outer root sheath, hair follicle stem cell areas, and the pilosebaceous ducts showed higher expression of the hBDs when compared to the inner compartments, such as the proximal outer and inner root sheaths, along with the hair follicle bulb (174). The authors suggested that hBD-2 protects the population of epidermal stem cells in the hair follicle from microbial invasion (175). Indeed, hBD-2 has the most potent antibacterial activity among hBDs (182). Furthermore, the expression of hBD-1 was only moderately induced in most acne lesions (comedones, papules, pustules) compared to non-lesional skin of the same patient and healthy back skin and pilosebaceous follicles of controls. In contrast, hBD-2 was moderately to strongly upregulated in all acne lesions. Overall, the expression of both hBDs in acne lesions was summarized as follows: hBD-1 - healthy follicular skin ≤ pustule ≤ comedo < papule; hBD-2 - healthy follicular skin ≤ comedo < papule < pustule, and interpreted as a secondary response to the perilesional infiltration by immune cells and secretion of pro-inflammatory cytokines, such as IL-1β (175). The expression of hBDs and other AMPs is a keratinocyte differentiation-dependent process. Therefore, keratinocytes may serve as sensors of abnormal colonization of PSUs by *C. acnes* acne-associated strains due to TLR2- and TLR4-induced secretion of hBD-2 and IL-8 (78, 175). This may be a critical inflammatory event in the development of AV, since both compounds are potent leukocytes and neutrophils chemoattractants (78). Specifically, hBDs can recruit dendritic cells (iDCs) and T cells via interaction with C-C motif chemokine receptors 2 and 6 (CCR2 and CCR6) (17, 19, 78, 79). Moreover, hBDs (and LL-37) can increase the release of mast cell inflammatory mediators and vascular permeability via activation of Mas-related G-protein-coupled receptor X (MrgX2) and G-protein-coupled receptor GPCR and mitogen-activated protein kinase (MAPK) signaling pathways (80). In addition, hDBs, due to arresting Th17 cells (but not Th1 or Th2 cells) on inflamed sites in a CCR6-dependent mechanism, may potentially contribute to the perpetuation of inflammation (180). Likewise, hBD-2 and hBD-3 via CCR6 play a positive and negative regulatory role in the development and proliferation of human effector CD4+ T cells, and they may shift their surface marker expression to regulatory phenotype (CD4+ CD25+) (183). In detail, the authors observed that co-culture with hBD-2 and hBD-3 increases or decreases CD4+ T cell proliferation after 72 or 96 hours, respectively (183). HBDs also utilize CCR6 to enhance the epidermal TJ barrier (**Figures 3**, **4**). Therefore, several either beneficial or potentially harmful pro-inflammatory effects can be exerted by hBDs in the CCR6-dependent mechanism, likely limiting its interaction with the canonical ligand, namely C-C motif chemokine ligand 20 (CCL20) (81). Upregulation of hBD-2 and CCR6 (along with LL-37) was also reported in psoriasis, highlighting the importance of the implicated CCR6-dependent signaling pathways in the pathomechanism of these dermatoses (82). Finally, hBDs (and LL-37) may also support anti-inflammatory responses, promoting keratinocyte migration and proliferation as well as wound healing via activation of epidermal growth factor receptor (EGFR) and signal transducer and activator of transcription (STAT) signaling pathways (80).

Individuals with insulin resistance are predisposed to AV, and it was suggested as an independent contributing factor in AV development that should be considered when diagnosing and treating acne (184, 185). Similarly, AV patients are at risk group for developing metabolic syndrome (MetS), i.e., a multisystem condition that raises diabetes, stroke, and cardiovascular disease risk (186). Increased blood glucose levels may contribute to AV development due to the stimulation of insulin release. Insulin, as a structural homolog of IGF-1, competes for its cellular receptor and, in turn, promotes IGF-1-mediated keratinocyte proliferation (83). Additionally, insulin enhances sebum production, upregulating androgen secretion. Insulin is a key regulator of hBD-1 expression through enhancing glucose uptake by the cells in a mechanism involving upregulation of the insulin-responsive glucose transporter (GLUT4) and the human sodium-glucose cotransporter (hSGLT1) expression (66). These observations may provide a link between metformin, a drug upregulating GLUT4 expression used to treat type 2 diabetes, and its application in AV therapy (84, 85). Hence, levels of hBDs, and possibly other AMPs, should be viewed in light of intracellular glucose concentration and the insulin transcriptional activity as vital explanatory variables of their expression in AV patients. For instance, hyperglycemia also decreases IL-6-mediated psoriasin (S100A7) expression in the urinary bladder, compromising uroepithelial barrier function and increasing susceptibility to *Escherichia coli* infection (86). Furthermore, Eicher et al. reported the induction of RNase 7 production in uroepithelial cells by insulin (via PI3K/Akt signaling pathway) as a protective mechanism against invasion by uropathogenic *E. coli* (77). Similarly, Yin et al. demonstrated that high glucose suppresses the wounding-induced upregulation of LL-37 in cultured human corneal epithelial cells (HCECs), and correlated wound healing activity of LL-37 with the activation of the heparin-binding EGF-like growth factor (HB-EGF)→EGFR→PI3K→Akt signaling pathway (87).

High-glucose and/or enhanced oxidative stress (OS) conditions promote advanced glycation endproducts (AGEs) accumulation, which contributes to several diabetic complications and AV progression (**Figure 4**) (71, 88, 89). In general, the toxicity of AGEs is mediated by their interaction with the transmembrane receptor for advanced glycation end products (RAGE), which increases OS and inflammatory processes by dysregulating multiple intracellular signaling pathways. Also, by enhancing OS, S100 proteins might be implicated in RAGE-induced inflammation (**Figure 4**) (90). The latter has been proposed as a biomarker index for AV activity and treatment monitoring (88). In fact, the therapeutic effectiveness of certain antimicrobial agents, such as tetracycline, macrolides, and metronidazole, is attributed mainly to their antioxidant effects, especially metronidazole as *C. acnes* is intrinsically resistant to this drug (70, 187). Similarly, FFAs, like linoleic acid, significantly suppress ROS (70). Accordingly, Xu et al. demonstrated *ex vivo* in cultured porcine and human corneas that high glucose delays corneal epithelial wound healing, likely in a ROS-dependent suppression of EGFR-PI3K/Akt signaling pathway (188). In detail, high glucose inhibited ROS-sensitive Akt phosphorylation. Thus, antioxidants, in combination with EGFR ligands, have been suggested as promising candidates for diabetic keratopathy treatments (188).

### 4.2 Impact of AMPs on epidermal TJs barrier function

HBDs, especially hBD-3, and other AMPs, like LL-37 and S100A7, may further influence AV pathogenesis by upregulating TJs proteins, as demonstrated based on transepithelial electrical resistance (TERs) measurement (19). For example, Kiatsurayanon et al. noted that hBD-3 (but not hBD-1, hBD-2, and hBD-4) increases the expression and localization of several claudins (CLDNs) at cell-cell borders of human keratinocytes (63). Additionally, the upregulation of (i) occludin and zonulin (ZO-1) by hBD-3 and (ii) occludin and mucin-2 (MUC2) by hBD-2, in human epithelial Caco-2 cells was reported by Fusco et al. (189).

Notably, the improvement of TJs function by hDB-3 is mediated via activation of PI3K, atypical protein kinase C (aPKC), glycogen synthase kinase-3 (GSK3), and Ras-related C3 botulinum toxin substrate 1 (Rac1), in CCR6-dependent signaling (63). As aforementioned, IGF-1 and insulin promote lipogenesis in sebaceous glands by modulating PI3K/Akt/mTOR/FoxO1 signaling pathway. PI3K also activates aPKC, which, in a complex with PAR (partition defective) proteins, i.e., PAR-3 and PAR-6 (PAR-3/PAR-6/aPKC), is a vital component of TJs, responsible for establishing and maintaining cell polarity (64, 65, 91, 92). aPKC is also involved in the IGF-I/insulin signaling pathways to regulate various metabolic processes, such as GLUT4-dependent glucose transport and lipogenesis. Moreover, it can modulate the inflammatory response via activation of NF-κB signaling pathways (91). Similarly, GSK3 controls glucose metabolism, controlling glycogen synthase (GS) in response to insulin stimulation. However, its role in AV might be more versatile. GSK3, due to its interaction with 40 targets and more than 500 substrates, is implicated in virtually every central biological process in the cell and is a potent regulator of inflammation (93–95). Subsequently, GSK3 contributes to several inflammatory and metabolic disorders, e.g., diabetes mellitus (93). Likewise, Rac1, a small GTPase, as a regulator of the actin cytoskeleton and other fundamental cellular processes, including cellular plasticity, migration, invasion, adhesion, proliferation, apoptosis, ROS production, and inflammation, is implicated in multiple pathological conditions (96, 97). Specifically, Rac1 coordinates a keratinocyte immune-cell crosstalk, and other AMPs, e.g., LL-37, also enhance its activity. Thus, it is essential for skin homeostasis, regulating (i) epidermal TJs barrier function, (ii) wound re-epithelialization, and (iii) inflammation (98). Rac1’s contribution to AV pathogenesis might be particularly associated with (i) maintaining hair follicle integrity and (ii) regulation of actin reorganization in insulin-induced recruitment of GLUT4, i.e., a mechanism controlling the expression of hBD-1 (66).

### 4.3 Role of specific AMPs (LL-37, LCN2, S100 proteins, SLPI, and AMP-IBP5) in AV

In human skin, LL-37 is primarily produced by keratinocytes (and sebocytes) in both constitutive and inducible mechanisms. It is crucial for maintaining skin barrier homeostasis due to direct antimicrobial activity and also an acceleration of the skin regeneration processes and wound healing process through the promotion of cell migration and proliferation as well as angiogenesis (99, 190). LL-37 is proteolytically processed into smaller peptides, such as RK-31 and KS-30, with enhanced activity against specific microbes, e.g., staphylococcal and candidal species, and acting synergistically with other AMPs, such as hBD-2. LL-37 is a well-known DAMP agent that regulates the immune surveillance system via interaction with multiple cellular receptors, including TLR4, EGFR, MrgX2, glyceraldehyde-3-phosphate dehydrogenase (GAPDH), and formyl-peptide-receptor-like-1 (FPRL-1) (191, 192). For instance, it chemoattracts neutrophils, monocytes, and T lymphocytes by binding to the FPRL-1 (192). In addition, LL-37 induces calcium (Ca^2+^) mobilization in cells through purinergic receptor P2X_7_, the process that controls cell migration (193). Moreover, LL-37 is a biased agonist of IGF-1R, i.e., an agonist that stabilizes distinct receptor active states, activating only specific downstream signaling cascades, which preferentially promotes the phosphorylation of ERK1/2 over Akt (100). The pro-inflammatory actions of LL-37 include decreased expression of IL-10, increased expression of IL-1β, IL-18, mast cell degranulation, and release of inflammatory mediators. In contrast, inhibition of interferon-induced protein formation AIM2, tumor necrosis factor TNF-α, IL-12, and IL-4 are central mechanisms behind its anti-inflammatory properties, mediated by neutralization of bacterial PAMPs, such as LPS, or LTAs (194).

Furthermore, like hBDs, LL-37 contributes to cutaneous immunity by improving the TJs barrier function and keratinocyte differentiation (99). LL-37 can increase in epidermal keratinocyte the mRNA and protein levels of numerous TJ proteins, including claudin-1, 3, 4, 7 and occluding, by the activation of the aPKC, Rac1, GSK-3, and PI3K signaling pathways (99). Also, multiple keratinocyte differentiation markers, such as filaggrin, involucrin, keratin 1, keratin 10, and transglutaminase 1 (TGM1), but not loricrin or transglutaminase 3 (TGM3), are markedly induced by LL-37 (78, 79, 99). Interestingly, the LL-37-mediated improvement of the TJ barrier appears to be connected with its ability to activate autophagy, i.e., a process associated with skin diseases manifested by a defective epidermal barrier (101, 193). Additionally, in psoriasis, LL-37 suppresses keratinocyte apoptosis via upregulation of apoptosis-related genes, such as cyclooxygenase- 2 (COX-2), an inhibitor of apoptosis-2 (IAP-2) (195). LL-37 anti-apoptotic activity may also contribute to AV pathogenesis, as only acne-associated *C. acnes* isolates induce keratinocyte/sebocyte proliferation and differentiation (78, 79). Similarly, the anti-inflammatory role of LL-37 associated with blocking the activation of the DNA-sensing inflammasomes might be a common trait for psoriasis and AV (14, 194).

Although LL-37 can directly kill *C. acnes*, the bactericidal concentration (4 μM) is 100 times higher than its amount in extracts from sebocytes (0.038 μM) (196). However, its combination with psoriasin (S100A7), at a concentration of 10 μg/mL, other overexpressed in acne lesions AMP, reduces its *C. acnes* killing concentration to 0.5 μM (195).

Psoriasin, named for its discovery in psoriatic patients, belongs to the vertebrate-specific and Ca^2+^-binding S100 family proteins (197, 198). The S100 family involves 25 proteins implicated with various intracellular and extracellular functions. Intracellularly, S100 proteins are responsible for (i) calcium homeostasis, (ii) energy metabolism, (iii) regulation of cell cytoskeleton as well as (iv) proliferation, differentiation, and apoptosis via interacting with multiple nuclear proteins (198, 199). Extracellularly, S100 proteins as signal molecules modulate the inflammatory response and act as AMPs (200). The former action is mediated via interaction with multiple cellular receptors (201). For instance, calprotectin (the complex of S100A8 and S100A9) as an agonist for TLR4 and RAGE acts as a DAMP molecule in various inflammatory responses and serves as a biomarker in several immunomodulatory, antiproliferative, and infectious diseases (202, 203). Recently, a significant positive correlation was also reported between serum calprotectin levels and acne severity and duration (204, 205). In the skin, S100 proteins are expressed by keratinocytes, sebaceous glands, and hair follicles and regulate epidermal differentiation and proliferation (102, 206, 207). In fact, S100 protein genes are located in the epidermal maturation region (Epidermal Differentiation Complex; human chromosome 1q21), involving genes crucial for epidermis maturation, such as involucrin, filaggrin, trichoyalin, and repetin (208). The production of S100A proteins is stimulated by inflammatory cytokines (IL-1, IL-17, and TNFα) and in response to infection; hence, they serve as markers in several dermatoses manifested by inflammation and keratinocyte hyperproliferation (17, 102, 207, 209).

The AMP function has been identified for psoriasin (S100A7), calgranulin A (S100A8), calgranulin B (S100A9), calgranulin C (S100A12), and koebnerisin (S100A15, S100A7A) (198, 200, 207), and it associated with their Zn^2+^ sequestration (172, 210). Neutrophils extensively express and release S100A8/A9 (calprotectin) and calgranulin C (S100A12) during infection, which account for ~50% of their total cytoplasmic proteins (201). However, Zn^2+^ sequestration by S100 is markedly restricted by low pH. Therefore, Wang et al. suggested that Ca^2+^ binding is a mechanism enhancing the antimicrobial action of S100 proteins (S100A12 and S100A8/A9), due to conformational changes which increase their affinity to Zn^2+^ even under the physiologically relevant sub-neutral pH conditions (between pH 5.5 – 6.0) expected for activated neutrophils (210). Furthermore, S100A8/S100A9 tetramers are produced at high extracellular Ca^2+^ concentrations, which act as an autoinhibitory mechanism that modulates S100A8/9 biological activity exerted by TLR4 (201).

Also, TJs barrier formation is affected by S100 proteins in a Ca^2+^-dependent mechanism (211). For example, psoriasin (S100A7) increases the expression of keratinocyte differentiation markers (filaggrin, involucrin, keratin 1, keratin 2, loricrin, TGM1, and TGM3). Moreover, it upregulates the skin’s TJ proteins (claudin-1, claudin-3, claudin-4, claudin-7, claudin-9, claudin-14, and occluding) via GSK-3 and MAPK pathways. Subsequently, psoriasin (S100A7) promotes the accumulation of controlled by GSK-3 components of adherens junctions - β-catenin and E-cadherin at cell-cell contacts (212). Therefore, depletion of Ca^2+^ reversibly disturbs the assembly of TJs proteins, such as occludin, claudin-1, claudin-4, or E-cadherin, and, in consequence, weakens the permeability barrier (211).

Furthermore, psoriasin (S100A7) activates cellular retinoic acid-binding protein 2 (CRABP-II), which regulates human skin cell proliferation and retinoic acid (RA)-mediated differentiation (102, 103). Levels of retinoids (vitamin A derivatives) and vitamin D are vital for epidermal cell development; thus, the above observations may explain the effectiveness of isotretinoin and calcipotriol as AV topical treatment, which upregulate and downregulate psoriasin’s (and koebnerisin’s) expression (17, 74, 75, 213). Moreover, in AV, psoriasin (S100A7) is upregulated in sebaceous glands and acne lesions, acting as a potent and selective chemotactic inflammatory factor for CD4+ T lymphocytes and neutrophils (214).

Interestingly, according to the transcriptomic study by Kelhälä et al. koebnerisin (S100A15) was the most among 509 overexpressed genes in AV patients (21). Similar to psoriasin, koebnerisin was initially discovered in psoriatic skin, and subsequently, it has also been recognized as a new factor in the pathophysiology of rosacea (208, 215). Indeed, in human keratinocytes, its expression is stimulated by cytokines typical for the acne inflammatory milieu, such as TNF-α, IFN-γ, and IL-1β, indicating that inflamed skin may trigger its production in the epidermis. In fact, psoriasin (S100A7) and koebnerisin (S100A15) evolved by gene duplication and share >90% sequence identity; hence, they are difficult to differentiate (208). Nevertheless, both S100 proteins show distinct tissue distribution, regulation, and function. The expression of psoriasin (S100A7) is limited to the granular/cornified layers of the interfollicular epidermis and hair follicles, whereas koebnerisin (S100A15) is also expressed by basal epidermal and dendritic cells. In psoriasis, IL-17A is a primary inducer of both S100 proteins, especially koebnerisin, whereas other Th17-related cytokines, such as TNF-α and IL-22, differently regulate their expression in epidermal keratinocytes (216). The presence of IL-17A-positive T cells and Th17-related cytokines (IL-1β, IL-6, TGF-β, IL23p19) is also characteristic of acne lesions (21). Nonetheless, both proteins are co-upregulated under related pathological conditions by similar epidermotropic and microbial pro-inflammatory mediators, indicating their collaboration in the inflammatory response, e.g., they act synergistically as DAMPs in leukocyte recruitment to exacerbate inflammation in psoriasis (208, 216). In addition, S100A15 has two splicing isoforms, i.e., short (S100A15-S) and long (S100A15-L), characterized by distinct regulation. For instance, S100A15-L exhibits a more pronounced response to pro-inflammatory Th1 cytokines, such as TNF-α, IFN-γ, and IL-1β, than S100A15-S, which may explain its ~25 times greater expression than S100A150-S in acne lesions, as suggested for psoriasis (178, 208, 216).

In acne lesions, S100 proteins may also be upregulated by lipocalin 2 (LCN2), which, promoting their keratinocyte secretion, aggravates psoriasiform skin inflammation in a Th17-dependent manner (104). LCN2, known as neutrophil gelatinase-associated lipocalin (NGAL), is an antimicrobial protein and multifunctional adipokine linked with insulin resistance, obesity, and atherosclerotic disease, and a potential biomarker for infection inflammation, ischemia, or kidney injury (217). In health, its skin and serum expression is low; however, keratinocytes and neutrophils overexpress LCN2 by TLR2- and TLR4-dependent signaling in various skin disorders, including AV (21, 218). Indeed, Al Hashimi et al. reported elevated LCN2 serum levels in AV patients compared to healthy controls (218, 219). Likewise, Watanabe et al. suggested LCN2 as an objective biomarker of acne symptoms, correlating its decreasing stratum corneum levels on the cheeks of patients with AV with symptom alleviation (203).

In sebocytes, LCN2 expression rises in response to *C. acnes* and IL-1β, as well as isotretinoin (220). For instance, in a study by Nelson et al., LCN2 was among the top genes most upregulated by isotretinoin in the skin and cultured human sebaceous gland cells (SEB-1 sebocytes) (221). Moreover, the authors found that LCN2 is substantially raised in the skin of AV patients during the first week of isotretinoin treatment but not eight weeks later (222). Since isotretinoin reduces sebum production through partly controlled by LCN2 induction of sebocyte apoptosis, an isotretinoin-induced increase of LCN2 precedes a decrease in sebum and a reduction in *C. acnes* abundance (220).

The antimicrobial activity of LCN2 activity is mediated by the sequestration of bacterial siderophores, i.e., iron-binding proteins. Hence, it acts with lactoferrin (LF), a potent iron chelator and broad-spectrum AMP, to restrict this essential element and impede bacterial growth. Indeed, LF can suppress *C. acnes*-induced inflammation in both *in vitro* and animal model studies (223). Lactoferrin, also known as lactotransferrin (Ltf), is an 80-kDa iron-binding glycoprotein of the transferrin family with multiple biological functions, including antimicrobial, immunomodulatory, anti-inflammatory, antioxidant, and enzymatic activities. Along with lysozyme, LL-37, and α-defensin-1, LF is a component of neutrophil granules, and it is secreted into numerous body fluids, including sweat (105). Therefore, LF has excellent potential as a topical or oral treatment for skin infections and dermatoses (105). For instance, in the study by Chan et al., oral administration of LF with vitamin E and zinc significantly reduced acne lesions in patients with mild to moderate AV in a randomized, double-blind, placebo-controlled trial involving 168 subjects (106). Although the mechanism of action for orally administered LF is uncertain, its ingestion is safe and (i) promotes oral and intestinal homeostasis, (ii) regulates glucose and lipid metabolism, (iii) reduces systemic inflammation, and (iv) iron absorption and balance (107). Therefore, it is a promising nontoxic adjuvant for the long-term prevention of metabolic illnesses, including insulin resistance, T2D, and metabolic syndrome (107).

Furthermore, LCN2 significantly influences intestinal and metabolic inflammation (217). It is overexpressed in individuals with type 2 diabetes mellitus (T2DM), obesity, or nonalcoholic steatohepatitis (NASH). Accordingly, LCN2 regulates the gut microbiota composition and intestinal permeability, i.e., factors that may lead to systemic inflammation, insulin resistance, metabolic syndrome, and related characteristics (217). Therefore, LF and LCN2 may represent AMPs linking intestinal (gut dysbiosis) and metabolic inflammation with AV, e.g., via the gut-brain-skin axis, emotional stresses, and neuroinflammation. The latter is controlled by certain neuropeptides possessing also antimicrobial and immunomodulatory activities, such as substance P and chromogranin B (secretogranin-I). For instance, a positive correlation between stress scale and serum level of substance P was reported in AV patients, and acne skin is highly innervated due to the abundance of SP-containing nerves (224–227). It also stimulates sebaceous gland growth and differentiation, increases lipid synthesis in sebocytes, promotes mast cell proliferation and degranulation, and the release of pro-inflammatory cytokines (224, 225, 227). Thus, SP may bridge AV pathomechanism with its neurogenic and psychogenic aspects (225, 226). However, the overexpression of substance P and chromogranin B is not universally recognized in acne skin (178). Recently, Kwiecinska et al. revealed a novel, SLPI-mediated mechanism responsible for maintaining skin homeostasis through a nerve-reflex arc, preventing excessive skin dryness in psoriasis and possibly also in other dermatoses with compromised skin barrier function (228). SLPI is a ~12 kDa cationic AMP protein and an essential regulator of innate and adaptive immunity, anti-inflammatory properties in allergy and autoimmunity, as well as a component of tissue regeneration programs (229). Like LCN2, SLPI may be responsible for the interplay between microbiota and epithelial cells, regulating the threshold for epithelial activation and microbial signals (229).

Another AMP that connects AV with its metabolic background could be AMP-IBP5, a 22 antimicrobial peptide generated from insulin-like growth factor-binding protein 5 (IGFBP-5). This 22-amino acid peptide shows antibacterial activity even better than LL-37 or hBD-2. In addition, it stimulates various keratinocyte and fibroblast functions via the receptor low-density lipoprotein receptor-related protein-1 (LRP1) and MrgX1-X4 receptors (108, 230–232). In addition, AMP-IBP5 suppresses the expression of Th2 cytokines such as IL-4, IL-13, IL-31, IL-33, and thymic stromal lymphopoietin (TSLP), stimulates the production of IL-8 and VEGF (108, 232). Furthermore, AMP-IBP5 improves the skin’s barrier function by upregulating and distributing TJs proteins, such as claudin-1, -4 and -7, occluding and ZO-1, through aPKC and Rac1 pathways (108). AMP-IBP5, unlike other AMPs, such as hBDs, LL-37, and S100A7, is downregulated in psoriatic skin tissues (109). Furthermore, AMP-IBP5 mitigates the harmful effects of the high glucose (HG) environment on keratinocyte proliferation and migration, as well as accelerates delayed angiogenesis and wound healing in diabetic mice via the EGFR, STAT, and MAPK pathways. Therefore, AMP-IBP5 may also be implicated in AV pathomechanism due to its protective effect against glucotoxicity (110).

The MAPK metabolic pathway is also activated by dermcidin (DCD), stimulating keratinocytes to generate cytokines and chemokines (19, 111, 112). In contrast to other AMPs, DCD is only constitutively expressed, i.e., regardless of the inflammatory conditions, in the eccrine sweat glands and secreted with sweat on the epidermal surface, followed by its proteolytic cleaving into active DCD-1 and DCD-1L (233, 234). Bactericidal concentrations of DCD against 68% and 83% of *C. acnes* isolates were estimated at 50 μg/mL and 270 μg/mL, respectively (235). Furthermore, Nakano et al. found reduced DCD levels in sweat from AV patients (median 9.8 μg/ml, range 6.9–95.3) compared to healthy volunteers (median 136.7 μg/ml, range 45.4–201.6 μg/ml) (235). Hence, DCD deficiency in the sweat of AV patients may allow *C. acnes* to colonize and multiply in the pilosebaceous unit, contributing to AV development (235).

Finally, skin microbiota-produced AMPs might also be implicated in AV pathogenesis as competitive exclusion or immunomodulatory factors, as in other dysbiotic disorders (236, 237). **Figure 4** summarizes the relationship between AMPs and the modulation of epidermal TJs barrier function in the context of AV metabolic background.

## 5 Development of anti-acne AMPs

Anti-acne therapeutic potential of human, non-human, semi-, and synthetic AMPs, associated with their antibacterial and anti-inflammatory activities, have been reported in several studies (238). For example, NAI003 peptide has completed a phase 1 clinical trial as an AV topical treatment candidate (EudraCT No. 2005-005531-99; https://www.clinicaltrialsregister.eu/) (239). NAI003 is a protein synthesis inhibitor targeting elongation factor Tu (EF-Tu) derived from *Planobispora rosea*-produced thiopeptide GE2270A, with a potent, selective action against *C. acnes* (MIC range 0.007 - 0.25 μg/mL) but not to other skin commensals, such as staphylococci (239). Also, the anti-acne activity of a synthetic lipohexapeptide HB-1345 and omiganan (CLS001/MBI 594AN, MBI 226 or MX 226), a 12 amino acid indolicidin-derivative, was assessed in clinical trials (ClinicalTrials.gov ID: NCT02571998; NCT00211523; NCT00211497) (240–243). Briefly, in a six-week, randomized, double-blind phase IIa study involving 75 subjects with facial AV omiganan reduced inflammatory lesions (papules and pustules; 39% vs. 21% reduction) in mild to moderate AV patients and non-inflammatory lesions (comedones; (10% *vs*. 25% reduction) compared to the placebo group, and improved physician’s Global Severity Assessment scores. However, in a longer, 12-week phase IIb randomized, double-blind, involving 241 participants, no statistically significant differences were noted beyond six weeks between the groups (242).

A reduction of acne severity after 12 weeks of topical treatment was also reported for a combination of 20 granulysin-derived peptides (GDP 20) in a study involving 30 AV patients (244). Similarly, combined therapy of GDP-20 with isotretinoin was superior over low-dose systemic isotretinoin alone in treating patients with mild-to-moderate AV (245). Furthermore, McInturff et al. designed five GDPs, and one of them, the D-type amino acid of peptide D-31–50v44w, effectively killed *C. acnes in vitro*, either in the growth media and in sebaceous microcomedome extracts as well as decreased *C. acnes*-stimulated production of cytokines and chemokines (246). Granulysin is a unique AMP since it is released by T cells instead of epithelial cells in the skin; hence, it serves as the adaptive immune agent rather than the innate immune system. T cells in early and late acne lesions imply granulysin’s importance for acne pathogenesis (246). Indeed, the presence of IL-17A-positive T cells and Th17-related cytokines in acne lesions suggests that the Th17 pathway is activated and may be crucial to the disease process (21).

In addition, anti-*C. acnes* and anti-inflammatory properties of non-human AMPs were investigated in several studies. For instance, Popovic et al. reported inhibition of *C. acnes* growth (MIC = 3-12.5 μM) and stimulation of anti-inflammatory cytokines (IL-10, TGF-β, and IL-4) production in peripheral blood mononuclear (PBM) cells by five frog skin-derived antimicrobial peptides ([D4k]ascaphin-8, [G4K]XT-7, [T5k]temporin-DRa, brevinin-2GU, and B2RP-ERa) (247). Moreover, modulation of adaptive immune defense associated with decreased T cell responses in favor of the protective function of Th2 cells, e.g., via suppression of pro-inflammatory IL-12 and IFN-γ cytokines by IL-10, appears to be an attractive trait of these AMPs (247). Accordingly, Ryu et al. showed that P5, a synthetic hybrid of cecropin A/magainin 2, efficiently kills *C. acnes* (MBC = 0.2 μM) and reduces the expression of pro-inflammatory cytokines IL-8 and TNF-α in *C. acnes*-treated human keratinocytes, likely via neutralization of its lipoteichoic acid, and has no cytotoxicity to skin cells (248). Likewise, Han et al. showed anti-*C. acnes* and anti-inflammatory activity of CEN1HC-Br, a 28 amino acid peptide isolated from the green sea urchin (249). In general, CEN1HC-Br was more active than clindamycin against 15 clinical *C. acnes* isolates with MIC range from 0.125 µg/mL to 32 µg/mL. In rats, CEN1HC-Br and clindamycin reduced *C. acnes*-induced ear swelling and the level of several pro-inflammatory factors (IL-8, TNF-α, MMP-2, and TLR2). However, only CEN1HC-Br significantly reduced in a TLR-dependent mechanism the expression of several pro-inflammatory cytokines, such as IL-12p40, IL-6, IL-1β, and TNF-α, in monocytes (249). Similarly, Bombinin-like peptide 7 (BLP-7) from *Bombina orientalis* has been shown to inhibit *C. acnes* growth (MIC = 5 μM) and to suppress the production of IL-8 and granulocyte-macrophage colony-stimulating factor (GM-CSF) by normal human epidermal keratinocytes (NHEKs) co-cultured with *C. acnes* (250). Additionally, in the rat ear edema model, BLP-7 efficiently reduced *C. acnes*-induced skin inflammation compared to the controls (250). Moronecidin, a 22 amino acid antimicrobial peptide derived from hybrid striped bass, was found to reduce *C. acnes*-induced inflammation in a rat model by these authors, and its MIC value against *C. acnes* was estimated at 10 μM (250). Furthermore, the study by Lee et al. revealed that melittin, an AMP isolated from honey bee venom, may suppress inflammatory cytokines, particularly TNF-a and IL-1β, by modulating NF-kB and AP-1 transcription factors, significantly reducing heat-killed *C. acnes*-induced inflammatory responses in keratinocytes. In addition, when administered intradermally to mice’s ears, melittin substantially reduced swelling and granulomatous responses as opposed to ears injected exclusively with living *C. acnes* (251). Likewise, a study by Wang et al. found that cathelicidin-BF, produced from snake Bungarus fasciatus, venom, has significant antibacterial action against *C. acnes* in an experimental mice skin colonization model. Cathelicidin-BF MIC values against two *C. acnes* strains (ATCC 6919 and ACTC 11827) were lower than those obtained for LL-37 and clindamycin (1.3 μM *vs* 2.2 μM *vs* 5.2 μM). Furthermore, in human monocytic cells, cathelicidin-BF significantly reduced secretion of the pro-inflammatory factors (TNF-a, IL-8, IL-1β, and MCP-1) as well as O^2.−^ production by human HaCaT keratinocyte cells triggered by *C. acnes*. The anti-inflammatory action was also confirmed *in vivo* with *C. acnes*-induced mice ear swelling and granulomatous inflammation (252). Anti-oxidant, anti-inflammatory, and anti-*C. acnes* (MIC = 400 µg/mL; MBC = 600 µg/mL) properties characterize also extracellular peptides (YTCY-Eps) isolated from *Weizmannia coagulans* strain YTCY, a probiotic gram-positive rod from in the family Bacillacea (253). These 9 - 18 amino acids peptides can *in vitro* reduce *C. acnes*-induced ROS level ~3 times and downregulated expression of inflammatory cytokines, chemokines, and MMPs genes by diminishing activation of TLR2 by *C. acnes* and subsequent NF-kB and MAPK/AP-1 signaling pathways. YTCY-Eps also reduced the expression of inflammatory cytokines and MMPs, as well as improved keratinization, on the rabbit ear acne model, suggesting their application as a potential anti-acne raw material in cosmetics (253). A relatively high MIC value - 200 μg/mL against *C. acnes* strain BCRC #10723 was also reported for a synthetic, 21 amino acids in length, antimicrobial peptide derived from the marine organism *Epinephelus coioides* epinecidin-1 by Pan et al. (254).

Synthetic or designed antimicrobial peptides (dAMPs), i.e., engineering analogs of naturally occurring AMPs characterized by a reduced risk of developing bacterial resistance, represent another approach to developing anti-acne AMPs (255–257). For example, Zhang et al. designed a 15 amino acid residues peptide named LZ1 characterized by potent, four times lower compared to clindamycin, antimicrobial activity against *C. acnes* (MIC = 0.6 µg/mL) and staphylococci (MIC values from 2.3 to 4.7 µg/mL), with little cytotoxic and hemolytic activity (255). Additionally, in the mice skin colonization model, ear swelling, inflammatory cell infiltration, and *C. acnes* colonization were significantly reduced by LZ1 by inhibiting the secretion of pro-inflammatory cytokines, IL-1β, and TNF-α (255). Similarly, Woodburn and colleagues developed five dAMPs (RP444, RP551, RP554, RP556, and RP557) with potent *in vitro* anti-*C. acnes*. One of them, RP556, as a topical agent (5 mg/mL), was successfully used to treat intradermal murine infection caused by multidrug-resistant *C. acnes* (256). Finally, Dong et al., using a series of deep learning (DL) models trained toward the prediction of antimicrobial and hemolytic activity, designed a set of 42 novel linear peptides. Five of them (14-15 amino acid residues) exhibited high potency (MIC = 2–4 µg/mL) and selectivity against *C. acnes* without simultaneous hemolytic and cytotoxic action (257).

**Figure 8:**
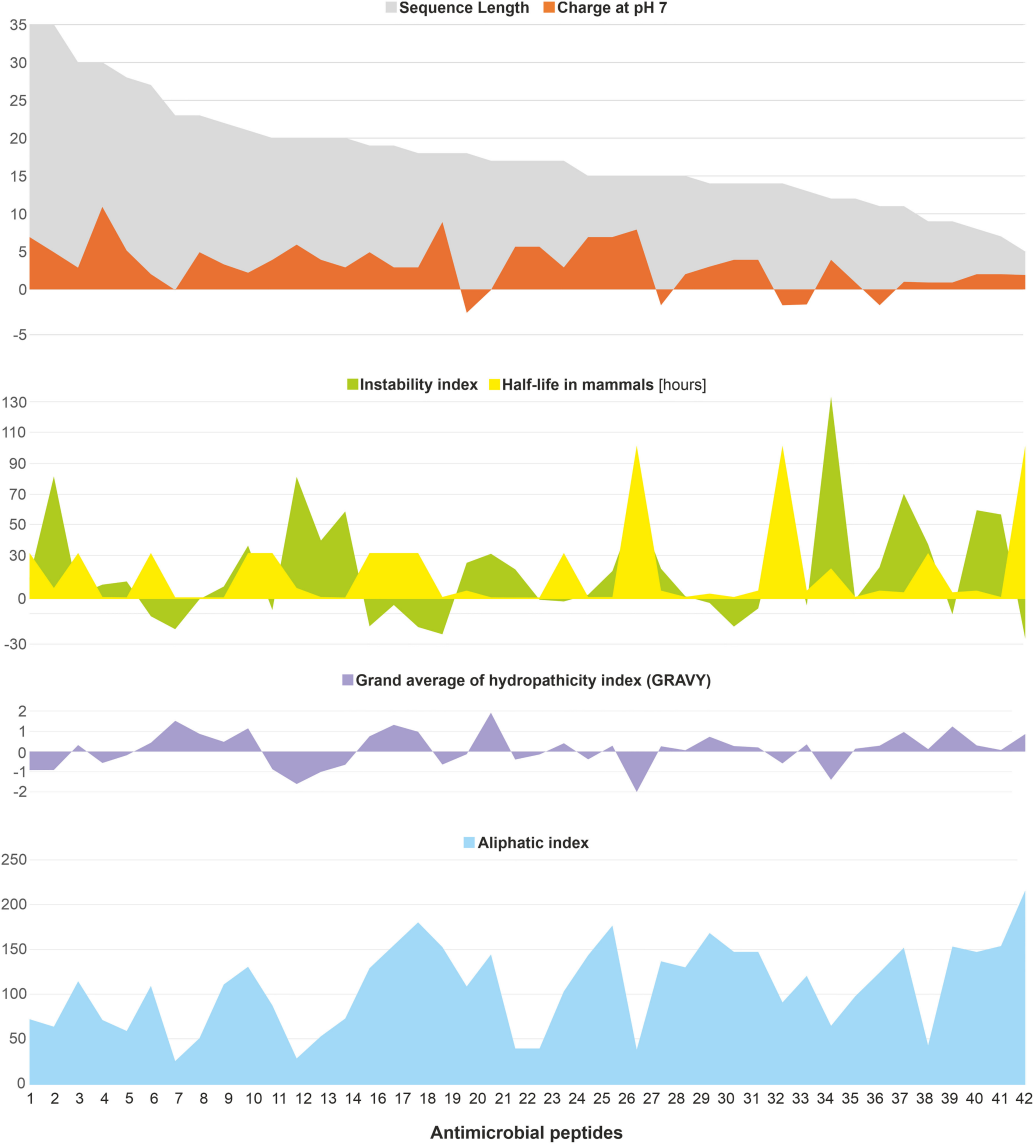
The physicochemical parameters of the developed anti-acne AMPs (n=42) discussed in the text; the graphs were created based on the AMPs parameters calculated using the ProtParam tool, using the website (https://web.expasy.org/protparam/), additional information included in **Supplementary Table S1**. The information in the figure summarizes the results of published experimental work (21, 238–257).

To summarize, the developed anti-acne AMPs represent a diverse group of either cationic and anionic peptides (charge at pH 7.0 ranging from -3.1 to +10.9), with length ranging from 5 to 35 residues, and various physicochemical properties, such as aliphatic index (range from 26.1 to 214.0), Grand average of hydropathicity index) (range from -1.9 to 1.824), and instability index (range from -26.0 to 131.9) (**Figure 8**; **Supplementary Table S1**). Nonetheless, several AMPs share specific amino acid motifs with human endogenous AMPs, including hBDs, LL-37, dermcidin, RNAse 7, or granulysin, which may explain their anti-acne properties (**Table 1**). For instance, LL-37 antimicrobial and immunomodulatory activities are linked to amino acid residues 13-32 and 17-29, respectively (258). Accordingly, AMP-29 shares the KI(I/G)K motif with LL-37, a part of its ‘antimicrobial’ region.

**Table 1:** Amino acid sequence motifs shared by AMPs developed to target *C. acnes* (highlighted in bold), i.e., AMP-29, AMP-31, and AMP-33 (257); P5 (248); [G4K]XT-7 and Brevinin-2GU (247); Cathelicidin-BF (252); RPM556 and RMP557 (256); YTCY_B (253), and human endogenous AMPs.

AMP	Amino acid sequence	Motif
**AMP-31**	KILG**KLLK**WASKIW	**KLL(E/K)**
**P5**	KWK**KLLK**KPLLK**KLLK**KL	
**[D4k]ascaphin-8**	GFK**KLLK**GAAKALVKTVLF	
**Hs04**	LFNNYITAAL**KLLE**KLYKV	
**hPF4**	EAEEDGDLQCLCVKTTSQVRPRHITSLEVIKAGPHCPTAQLIATLKNGRKICLDL QAPLYKKIIK**KLLE**S	
**IL-26**	MLVNFILRCGLLLVTLSLAIAKHKQSSFTKSCYPRGTLSQAVDALYIKAAWLKATIPE DRIKNIRLLKKKTKKQFMKNCQFQEQLLSFFMEDVFGQLQLQGCKKIRFVEDFHSLRQKLSHCISCASSAREMKSITRMKRIFYRIGNKGIYKAISELDILLSWIK**KLLE**SSQ	
**P5**	KWKKLLKK**PLLK**KLLKKL	**(P/N)LLK**
**[G4K]XT-7**	GLLK**PLLK**IAAKVGSNLL	
**CGA-N46**	PMPVSQECFETLRGHERILSILRHQ**NLLK**ELQDLALQGAKERAHQQ	
**sfTSLP**	MFAMKTKAALAIWCPGYSETQINATQAMKKRRKRKVTTNKCLEQVSQLQGL WRRFNR**PLLK**QQ	
**CXCL10**	VPLSRTVRCTCISISNQPVNPRSLEKLEIIPASQFCPRVEIIATMKKKGEKRCL NPESKAIK**NLLK**AVSKERSKRSP	
**AMP-33**	LSKWLK**KLGK**LLAG	**KLGK**
**Dermcidin**	SSLLEKGLDGAKKAVGGLG**KLGK**DAVEDLESVGKGAVHDVKDVLDSV	
**Cathelicidin-BF**	KFFR**KLKK**SVKKRAKEFFKKPRVIGVSIPF	**K(L/V/Q)KK**
**SPINK9-v4**	KQMVDCSHYKKLPPGQQRFCHHMYDPICGSDGKTYKNDCFFCS**KVKK**TDGT LKFVHFGKC	
**SPINK9-v1**	KQTKQMVDCSHYKKLPPGQQRFCHHMYDPICGSDGKTYKNDCFFCS**KVKK**T DGTLKFVHFGKC	
**Eotaxin-2**	VVIPSPCCMFFVSKRIPENRVVSYQLSSRSTCLKAGVIFTTKKGQQSCGDPKQ EWVQRYMKNLDA**KQKK**ASPRARAVA	
**Granulysin**	GRDYRTCLTIVQ**KLKK**MVDKPTQRSVSNAATRVCRTGRSRWRDVCRNFMRR YQSRVTQGLVAGETAQQICEDLRLCIPSTGPL	
**S100A9**	MEDKMSQMESSIETIINIFHQYSVRLGHYDTLIQKEFKQLVQKELPNFLK**KQKK** NEAAINEIMEDLDTNVDKQLSFEEFIMLVARLTVASHEEMHNTAPPGQGHRHG PGYGKGGSGSCSGQGSPDQGSHDLGSHGHGHGHSHGGHGHSHGGHGHSH	
**RP556**	RWCFKVCYKGICY**KKCK**	**K(K/Q/R/C)CK**
**RP557**	RFCWKVCYKGICF**KKCK**	
**AMP-IBP5**	AVYLPNCDRKGFYKR**KQCK**PSR	
**hBD-1**	DHYNCVSSGGQCLYSACPIFTKIQGTCYRGKA**KCCK**	
**hBD-2**	PVTCLKSGAICHPVFCPRRYKQIGTCGLPGT**KCCK**KP	
**hBD-28**	ARLKKCFNKVTGYCR**KKCK**VGERYEIGCLSGKLCCAN	
**hBD-26**	WYVKKCLNDVGICK**KKCK**PEEMHVKNGWAMCGKGRDCCVPAD	
**hBD-126**	NWYVKKCLNDVGICK**KKCK**PEEMHVKNGWAMCGKQRDCCVPADRRANYPVF CVQTKTTRISTVTATTATTTLMMTTASMSSMAPTPVSPTG	
**hBD-118**	SGEKKCWNRSGHCR**KQCK**DGEAVKDTCKNLRACCIPSNEDHRRVPATSPTPL SDSTPGIIDDILTVRFTTDYFEVSSKKDMVEESEAGRGTETSLPNVHHSS	
**RNase 7**	KPKGMTSSQWFKIQHMQPSPQACNSAMKNINKHT**KRCK**DLNTFLHEPFSSVA ATCQTPKIACKNGDKNCHQSHGAVSLTMCKLTSGKYPNCRYKEKRQNKSYVV ACKPPQKKDSQQFHLVPVHLDRVL	
**Brevinin-2GU**	GVIIDTLKGAAKTVAAE**LLRK**AHCKLTNSC	**LLRK**
**Buforin I**	AGRGKQGGKVRAKAKTRSSRAGLQFPVGRVHR**LLRK**GNY	
**AMP-29**	KKIFKRIV**KIIK**RLL	**KI(I/D/G)K**
**Cathelicidin LL-37**	LLGDFFRKSKE**KIGK**EFKRIVQRIKDFLRNLVPRTES	
**hPF4**	EAEEDGDLQCLCVKTTSQVRPRHITSLEVIKAGPHCPTAQLIATLKNGRKICL DLQAPLYK**KIIK**KLLES	
**Psoriasin**	MSNTQAERSIIGMIDMFHKYTRRDD**KIDK**PSLLTMMKENFPNFLSACDKKGT NYLADVFEKKDKNEDKKIDFSEFLSLLGDIATDYHKQSHGAAPCSGGSQ	
**YTCY_B**	MAVKVGINGFG**RIGR**NV	**R(I/T/G)GR**
**hGAPDH**	GKVKVGVNGFG**RIGR**LVTRAAFNSGKVDIVA	
**HD-5**	ATCYC**RTGR**CATRESLSGVCEISGRLYRLCCR	
**hBD-3**	GIINTLQKYYCRV**RGGR**CAVLSCLPKEEQIGKCSTRGRKCCRRKK	
**nBD-4**	EFELDRICGYGTARCRKKCRSQEY**RIGR**CPNTYACCLRKWDESLLNRTKP	
**Granulysin**	GRDYRTCLTIVQKLKKMVDKPTQRSVSNAATRVC**RTGR**SRWRDVCRNFM RRYQSRVTQGLVAGETAQQICEDLRLCIPSTGPL	

Hs04, human unconventional myosin 1H protein (predicted, encrypted AMP); hPF4, human Platelet Factor 4 (CXCL4, CXC family; kinocidin); IL-26, (interleukin 26, kinocidin); CGA-N46, a fragment of human chromogranin A (neuropeptide); sfTSLP, short form Thymic stromal lymphopoietin isoform 2 (cytokine); CXCL10, IFN–inducible protein 10 (CXC family, kinocidin); SPINK9-v4, serine peptidase inhibitor variant 4); SPINK9-v1, serine peptidase inhibitor variant 1; AMP-IBP5, antimicrobial peptide derived from insulin–like growth factor (IGF)–binding protein; S100A9, S100 family of protein A9 (Calgranulin–B); hBD, human β-defenisn; HD5, human α-defensisn 5; hGAPDH, a fragment (2–31) of glyceraldehyde-3-phosphate dehydrogenase. The latter (n=160) were obtained from the APD3 database (Antimicrobial Peptide Database; https://aps.unmc.edu/; prompt: AMP Database Search→Source Organism: *Homo Sapiens*; last accessed 17.03.2024); the analysis was performed with ‘Pattern Discovery’ module and CLC Genomics Workbench v24 software. AMP-29, AMP-31, and AMP-33 (257); P5 (248); [G4K]XT-7 and Brevinin-2GU (247); Cathelicidin-BF (252); RPM556 and RMP557 (256); YTCY_B (253), and human endogenous AMPs.

## 6 Discussion

These observations undoubtedly identify AMPs as crucial factors in the pathomechanism of AV. However, the complicated network of interactions between AMPs and other host factors as well as skin microbiota, remains to be deciphered. In addition, significant variation in the intensity of AMPs expression among patients, even when matched for sex and age, and between different body regions and hair follicles indicate that individual factors, possibly genetic or environmental, must be considered in such investigations (175, 259). For instance, polymorphism in cutaneous androgen metabolism-regulated genes *HSD3B1* and *HSD17B3*, cytochrome P450 family genes (*CYP17* and *CYP19A1)*, and in genes involved in immune responses is correlated with a risk of developing AV (152, 259–262). It highlights the intricate nature of AV as a multifactorial disease, involving a network of reciprocal interactions between hormonal, metabolic, immunological, microbiological, genetic, and psycho-emotional factors, with AMPs as their central regulatory and ‘effector nodes.’ In particular, hBD-2, and psoriasin (S100A7), appear to be its ‘critical nodes’, involved either in active and healed (subclinical) stages of AV. Furthermore, lipocalin 2 (LCN2) and lactoferrin (LF) may constitute AMPs linking intestinal (gut dysbiosis) and metabolic inflammation with AV, e.g., via the gut-brain-skin axis, emotional stress, and neuroinflammation. Therefore, deciphering complex mechanisms behind their expression and activated cellular pathways is crucial to elucidate their role in AV development and progression. For instance, the importance of AMPs competing with the canonical ligands (and likely between each other) for several cellular receptors associated with immune (e.g., CCR6) and metabolic (e.g., IGF-1R) responses is noteworthy, as AMPs may function as their biased agonists.

Also, the role of AMPs in maintaining epidermal TJs homeostasis deserves further investigation. The skin’s permeability and antibacterial barriers are inextricably linked, as disturbance of the mechanical barrier induces the expression of AMPs while regeneration reduces their level (212, 263). Since the formation of TJs is a Ca^2+^-dependent process, the role of calcium-binding and highly elevated in acne lesions S100A proteins, such as psoriasin (S100A7), S100A8/A9 (calprotectin), S100A12 (calgranulin C), and koebnerisin (S100A15, S100A7A), appear to be fundamental. In this light, the increased expression of AMPs in AV may reflect a compensatory mechanism to protect the skin with an impaired permeability barrier via activation of core cellular proteins, such as PI3K, GSK-3, aPKC, and Rac1. Therefore, AMPs may be key determinants in developing and progressing acne-associated immune responses, skin barrier integrity, and metabolic factors, like insulin/IGF-1 and PI3K/Akt/mTOR/FoxO1 signaling pathways or high glucose levels. From this perspective, AMP-IBP5, an antimicrobial peptide derived from IGF-binding protein 5, with superior antibacterial activity over hBD-2 or LL-37, which reduces the expression of Th2-specific cytokines, mitigates adverse effects of glucotoxicity and accelerates angiogenesis and wound healing in diabetic mice, is a promising candidate for further researches in this area. However, the antibacterial activity of AMPs must be carefully balanced in future anti-AV therapies to target specifically acne-associated *C. acnes* strains or to promote the ‘healthy’ ones rather than indiscriminately eradicate all *C. acnes* populations.
