# Supplementary material for: Significance of host antimicrobial peptides in the pathogenesis and treatment of acne vulgaris
Source: Front Immunol. 2024 Dec 18;15:1502242. doi: 10.3389/fimmu.2024.1502242 (PMC11688235; doi:10.3389/fimmu.2024.1502242)
Supplement: Supplementary file 1 [file Table1.docx]

Supplementary Material

# Supplementary Figures and Tables

**Supplemental Table S1**. The physicochemical parameters of the developed anti-acne AMPs (n=42); the parameters were calculated using the ProtParam tool (<https://web.expasy.org/protparam/>).

| **No.** | **Name** | **Sequence Length** [aa] | **Charge**  **at pH 7** | **GRAVY*** | **Aliphatic index** | **Instability index** | **Half-life mammals**** [hrs] | **Sequence** |
| --- | --- | --- | --- | --- | --- | --- | --- | --- |
| 1 | Granulysin-1 | 35 | 6,9 | -0.871 | 72,286 | 13.95 | 30 | GRDYRTSLTIVQKLKKMVDKPTQRSVSNAATRVSR |
| 2 | Granulysin-2 | 35 | 4,9 | -0.866 | 64 | 79.91 | 7,2 | TGRSRWRDVSRNFMRRYQSRVIQGLVAGETAQQIS |
| 3 | brevinin-2GU | 30 | 2,9 | 0.303 | 114 | 2.78 | 30 | GVIIDTLKGAAKTVAAELLRKAHCKLTNSC |
| 4 | Cathelicidin-BF | 30 | 10,9 | -0.537 | 71,333 | 9.34 | 1,3 | KFFRKLKKSVKKRAKEFFKKPRVIGVSIPF |
| 5 | CEN1HC | 28 | 5,1 | -0.175 | 59,286 | 11.42 | 1,1 | FKKTFHKVSHAVKSGIHAGQRGCSALGF |
| 6 | BLP-7 | 27 | 2,0 | 0.415 | 108,889 | -11.30 | 30 | GIGGALLSAGKSALKGLAKGLAEHFAN |
| 7 | RP444 | 23 | -0,1 | 1.443 | 26,087 | -19.69 | 1,1 | FAOOFAOOFOOFAOOFAOFAFAF |
| 8 | RP551 | 23 | 4,9 | 0.830 | 51,304 | -0.18 | 1,1 | FIOKFAKOFKOFIOKFAKFAFAF |
| 9 | Moronecidin | 22 | 3,3 | 0.455 | 110,455 | 8.11 | 1,1 | FFHHIFRGIVHVGKTIHRLVTG |
| 10 | Epinecidin-1 | 21 | 2,2 | 1.090 | 130 | 34.78 | 30 | GFIFHIIKGLFHAGKMIHGLV |
| 11 | Granulysin-3 | 20 | 3,9 | -0.830 | 87,5 | -7.15 | 30 | GRDYRTSLTIVQKLKKMVDK |
| 12 | Granulysin-4 | 20 | 5,9 | -1.525 | 29 | 79.64 | 7,2 | TRVSRTGRSRWRDVSRNFMR |
| 13 | Granulysin-5 | 20 | 3,9 | -0.955 | 53,5 | 38.10 | 1,3 | KMVDKPTQRSVSNAATRVSR |
| 14 | Granulysin-6 | 20 | 2,9 | -0.620 | 73 | 57.09 | 1 | RNFMRRYQSRVIQGLVAGET |
| 15 | [D4k]ascaphin-8 | 19 | 4,9 | 0.716 | 128,421 | -17.83 | 30 | GFKKLLKGAAKALVKTVLF |
| 16 | B2RP-ERa | 19 | 2,9 | 1.253 | 153,684 | -3.93 | 30 | GVIKSVLKGVAKTVALGML |
| 17 | [G4K]XT-7 | 18 | 2,9 | 0.928 | 178,889 | -18.33 | 30 | GLLKPLLKIAAKVGSNLL |
| 18 | P5 | 18 | 8,9 | -0.611 | 151,667 | -23.04 | 1,3 | KWKKLLKKPLLKKLLKKL |
| 19 | YTCY_2 | 18 | -3,1 | -0.128 | 108,333 | 23.59 | 5,5 | LAEREPANLGWGDLGVDV |
| 20 | RP554 | 17 | -0,1 | 1.824 | 143,529 | 29.46 | 1,1 | FOLOAOIOVOLOAOIOL |
| 21 | RP556 | 17 | 5,6 | -0.376 | 40 | 19.28 | 1 | RWCFKVCYKGICYKKCK |
| 22 | RP557 | 17 | 5,6 | -0.135 | 40 | -0.48 | 1 | RFCWKVCYKGICFKKCK |
| 23 | YTCY_B | 17 | 2,9 | 0.388 | 102,941 | -1.59 | 30 | MAVKVGINGFGRIGRNV |
| 24 | AMP-12 | 15 | 6,9 | -0.367 | 142,667 | 2.77 | 1,3 | KKWRKVLKLIKRLVG |
| 25 | AMP-29 | 15 | 6,9 | 0.273 | 175,333 | 18.22 | 1,3 | KKIFKRIVKIIKRLL |
| 26 | LZ1 | 15 | 7,9 | -1.900 | 38,667 | 61.67 | 100 | VKRWKKWWRKWKKWV |
| 27 | YTCY_4 | 15 | -2,1 | 0.247 | 136 | 19.75 | 5,5 | LVAELGKNVTVEEVN |
| 28 | YTCY_C | 15 | 2,0 | 0.060 | 129,333 | 1.75 | 1,4 | NRVIVEGVNVVKKHA |
| 29 | [T5k]temporin-DRa | 14 | 3,0 | 0.693 | 167,143 | -2.56 | 3,5 | HFLGKLVNLAKKIL |
| 30 | AMP-31 | 14 | 3,9 | 0.257 | 146,429 | -18.01 | 1,3 | KILGKLLKWASKIW |
| 31 | AMP-33 | 14 | 3,9 | 0.193 | 146,429 | -6.16 | 5,5 | LSKWLKKLGKLLAG |
| 32 | Granulysin-7 | 14 | -2,1 | -0.557 | 90,714 | 58.93 | 100 | VAGETAQQISEDLR |
| 33 | YTCY_3 | 13 | -2,0 | 0.338 | 120 | -4.22 | 5,5 | LVLHGGTGIPEAD |
| 34 | Omiganan | 12 | 3,9 | -1.325 | 65 | 131.93 | 20 | ILRWPWWPWRRK |
| 35 | YTCY_E | 12 | 0,9 | 0.133 | 97,5 | -0.31 | 1,4 | NVFRAALGNPNV |
| 36 | YTCY_1 | 11 | -2,1 | 0.273 | 123,636 | 20.67 | 5,5 | LEEVGAGVEVK |
| 37 | YTCY_D | 11 | 1,0 | 0.918 | 150,909 | 68.62 | 4,4 | AALGTPVHILK |
| 38 | YTCY_A | 9 | 0,9 | 0.111 | 43,333 | 35.62 | 30 | GMAMRVPTP |
| 39 | YTCY_F | 9 | 0,9 | 1.178 | 152,222 | -9.98 | 4,4 | AISLGTAKI |
| 40 | APAP2-modified | 8 | 2,0 | 0.287 | 146,25 | 57.88 | 5,5 | LAIVGHKR |
| 41 | APAP1-modified | 7 | 2,0 | 0.071 | 152,857 | 55.14 | 1,3 | KRLHVIG |
| 42 | APAP3-modified | 5 | 1,9 | 0.820 | 214 | -25.96 | 100 | VRIKL |

* - Grand average of hydropathicity index (GRAVY)

** - mammalian reticulocytes (*in vitro*)
